# Accessing a Hidden Pathway to Supramolecular Toroid through Vibrational Strong Coupling

**DOI:** 10.1021/jacs.5c02960

**Published:** 2025-05-29

**Authors:** Shunsuke Imai, Takumi Hamada, Misa Nozaki, Takatoshi Fujita, Mariko Takahashi, Yasuhiko Fujita, Koji Harano, Hiroshi Uji-i, Atsuro Takai, Kenji Hirai

**Affiliations:** † Division of Photonics and Optical Science, Research Institute for Electronic Science (RIES), 12810 Hokkaido University, North 20 West 10, Kita Ward, Sapporo, Hokkaido 001-0020, Japan; ‡ Division of Information Science and Technology, Graduate School of Information Science and Technology, Hokkaido University, North 14 West 9, Kita Ward, Sapporo, Hokkaido 060-0814, Japan; § Molecular Design and Function Group, 52747 National Institute for Materials Science (NIMS), 1-2-1 Sengen, Tsukuba, Ibaraki 305-0047, Japan; ∥ Department of Materials Science and Engineering, Faculty of Pure and Applied Sciences, University of Tsukuba, 1-1-1 Tennodai, Tsukuba, Ibaraki 305-8577, Japan; ⊥ Institute for Quantum Life Science, National Institutes for Quantum Science and Technology, 4-9-1 Anagwa, Inage, Chiba 263-8555, Japan; # Research Institute for Sustainable Chemistry, 13508 National Institute of Advanced Industrial Science and Technology (AIST), Kagamiyama 3-11-32, Higashihiroshima, Hiroshima 739-0049, Japan; ¶ Center for Basic Research on Materials, National Institute for Materials Science (NIMS), 1-1 Namiki, Tsukuba, Ibaraki 305-0044, Japan; ∇ Research Center for Autonomous Systems Materialogy (ASMat), Institute of Integrated Research, Institute of Science Tokyo, 4259 Nagatsuda-cho, Midori-ku, Yokohama, Kanagawa 226-8501, Japan; ○ Department of Chemistry, 26657 KU Leuven, Celestijnenlaan 200F, Heverlee, Leuven 3001, Belgium; ⧫ Institute for Integrated Cell-Material Science (WPI-iCeMS), Kyoto University, Yoshida, Sakyo-ku, Kyoto 606-8317, Japan

## Abstract

Control over specific intermolecular interactions is crucial to the formation of unique supramolecular assemblies. Recently, vibrational strong coupling (VSC) has emerged as a new tool for manipulating these interactions. Although VSC shows promise for controlling molecular assembly, it has not yet demonstrated the capability to open a pathway for creating structures that are inaccessible by conventional assembly methods. Here, we used VSC to control the transformation process of a naphthalenediimide supramolecular polymer induced by a click reaction. The supramolecular polymers with reactive ethynyl groups undergo a transformation from long fibers to thick fibers upon induction by an amino-yne click reaction in the absence of VSC. Under VSC of the C–H stretch, the click reaction within supramolecular polymers is accelerated; no such acceleration occurs in the reaction of individual monomers, suggesting that the acceleration is due to changes in the assembled structures. Indeed, applying the VSC to the C–H stretch uniquely altered the morphological transformation process, leading to the formation of metastable toroids instead of thick fibers. Notably, the molecular assembly cannot be directed toward a toroidal structure without a VSC. Theoretical simulations suggested that slipped packing configurations in the supramolecular polymers form the curvature necessary for toroidal structures. The experimental results, supported by theoretical simulations, suggest that intermolecular interactions among naphthalenediimide molecules are modified under VSC, leading to a slipped packing configuration of the toroidal assembly. These findings link the VSC-induced modulation of intermolecular interactions to structural outcomes, establishing VSC as a tool for manipulating molecular assembly beyond traditional assembly methods.

## Graphical abstract

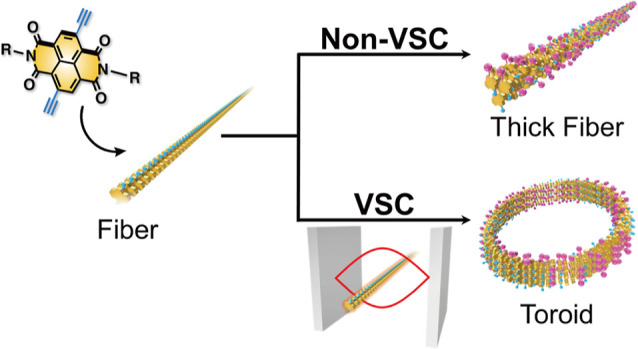

## Introduction

Molecules with specific intermolecular interactions can form intricate structures, as seen not only in biological systems such as proteins and the DNA helix but also in synthetic materials such as supramolecular polymers^1–5^ and porous materials.^6,7^ These interactions dictate the assembly process and ultimately influence the stability and functionality of the resulting structures. Historically, organic synthesis has enabled the introduction of specific functional groups into molecular components to tailor intermolecular interactions. In sharp contrast to conventional organic synthesis, vibrational strong coupling (VSC) has emerged as a new tool for manipulating intermolecular interactions.^8–13^

VSC was initially used to control chemical reactions,^14–17^ including organic and enzymatic reactions.^18,19^ Subsequently, the scope of VSC expanded to influence self-assembly, encompassing systems such as polymer assemblies,^20^ metal–organic frameworks,^21^ DNA origami,^22^ and supramolecules.^23^ Under VSC, assembly behaviors are likely affected by altered intermolecular interactions among solutes and solvent molecules. This mechanistic hypothesis has recently been supported by direct observations of changes in London dispersion forces induced by VSC.^24^ While VSC shows potential as a tool for controlling molecular assembly, the resulting structures are basically the same as those obtained through conventional assembly methods or depolymerized monomers. In other words, the VSC has yet to demonstrate its unique advantages as a tool for creating assemblies that are inaccessible through conventional assembly processes.

Meanwhile, there has been a flourishing development of supramolecular polymers that change their nanoscale assembled structures through chemical reactions.^25–30^ The transformation of assembled structures induced by chemical reactions suggests that the transformation process can fluctuate under additional stimuli, leading to the formation of new assembled structures. We envision that applying VSC to manipulate intermolecular interactions in such reactive supramolecular polymers will open new pathways for accessing metastable assembled structures that cannot be formed by conventional methods.

In this study, we employed supramolecular polymers incorporating amino-yne click reaction sites, which enable the transformation of assembled structures at room temperature without the need for a catalyst. By applying VSC during this transformation process induced by a click reaction within supramolecular polymers, we found that the supramolecular polymers can settle into previously inaccessible metastable states, leading to the formation of new assembled structures under VSC (Figure 1).

**Figure 1:**
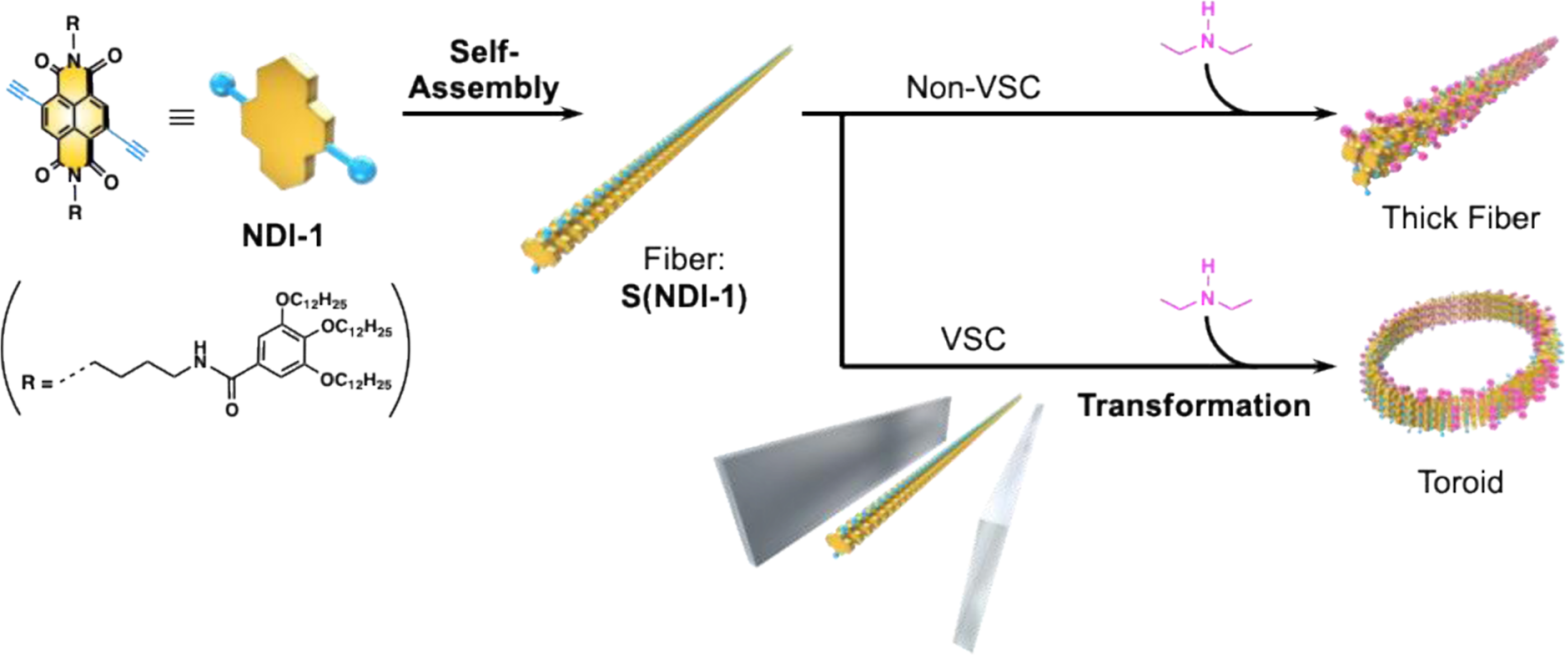
Schematic illustration of the transformation of supramolecular polymers in response to a click reaction. The assembly of **NDI-1** forms supramolecular polymers. While click reactions typically result in thick fibers, the process under VSC conditions led to the formation of toroids.

## Results and Discussion

### Click Reaction within Supramolecular Polymers under VSC

We have developed supramolecular polymers that undergo a transformation of their assembled structures induced by a catalyst-free click reaction.^31^ The molecule features a naphthalenediimide (NDI) core with ethynyl groups and long alkyl chains at the imide positions, denoted as **NDI-1** (Figure 1). Due to hydrogen bonding between amide groups, interactions between alkyl chains, and π–π interactions between the NDI core, **NDI-1** spontaneously assembles into supramolecular polymers, denoted as **S­(NDI-1)** (see Supporting Information for preparation details). Following fiber formation, the fibers can undergo an amino-yne click reaction between the ethynyl group and diethylamine (DEA) to afford an amine monoadduct quantitatively (Figure S1a). This process changes the interactions among NDIs, resulting in morphological changes of the supramolecular polymers. Because this transformation process is influenced by interactions among fibers and solvent molecules, the VSC offers a potential means to change the transformation.

**S­(NDI-1)** was dispersed in a methylcyclohexane (MCH) and toluene mixture (4:1 by volume; 2 mM in the monomer unit) and introduced into the Fabry–Perot (FP) cavity. The reflection mirrors were fabricated using indium tin oxide (ITO)-coated BaF_2_, as both ITO and BaF_2_ are transparent to visible light but reflective in the infrared (IR) range (Figure S2). This design enables the ITO-coated BaF_2_ mirrors to reflect IR light but allow real-time monitoring of the click reaction process in the visible light range.^32^ In contrast, Au and Al reflect and absorb visible light, which can distort the absorption peaks.^33^ Therefore, the ITO is a more suitable choice for this system (Figure S3).

The absorption of C–H stretching vibrations of the alkyl groups of **S­(NDI-1)** and the solvent molecules (MCH/toluene) was observed in the range of 2850 cm^–1^ and 2950 cm^–1^. By tuning the cavity mode to match the energy of the C–H stretching vibrations, two new peaks appeared at 3000 cm^–1^ and 2815 cm^–1^, corresponding to the formation of upper and lower polaritons. The observed Rabi splitting of these polaritonic states indicates the state of the VSC. A significant Rabi splitting of 185 cm^–1^ was observed, exceeding the full width at half-maximum of the cavity mode (45 cm^–1^) and the bare C–H stretching absorption (85 cm^–1^), further suggesting the VSC state (Figure 2).^34^

**Figure 2:**
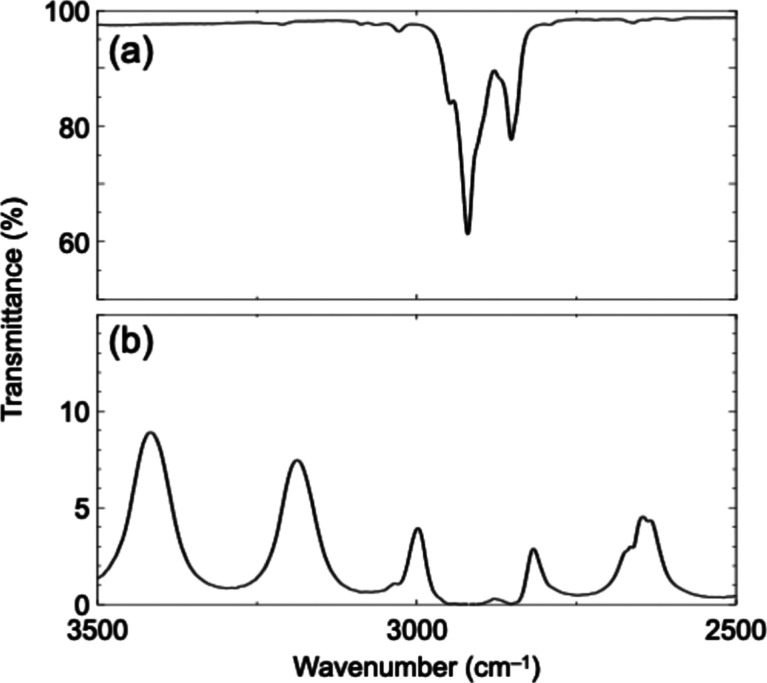
Fourier transform IR spectra of (a) MCH/toluene solution containing **S­(NDI-1)** and DEA and (b) solution introduced in an FP cavity. The cavity mode was tuned to 2908 cm^–1^ to achieve VSC.

**S­(NDI-1)** solution (2 mM in monomer unit) was mixed with a DEA solution (2 mM) and introduced into an FP cavity to carry out the click reaction. Due to the instrumental and solubility limitations, we fixed the concentration of **S­(NDI-1)** and DEA at 2 mM, but we confirmed that there was negligible concentration dependence on the resulting product and its morphologies. Typical UV–vis absorption derived from **S­(NDI-1)** was observed before and immediately after the initiation of the click reaction, indicating that no significant dissociation to monomeric **NDI-1** likely occurred under VSC conditions or by the addition of DEA. As the click reaction progressed, the π–π* transition band (∼446 nm) decreased, while a charge transfer band (∼600 nm) emerged due to the introduction of an electron-donating amino group adjacent to the electron-accepting NDI core (Figure S4). It should be noted that the product is an amine monoadduct only, judging from the UV–vis absorption and ^1^H NMR spectra (Figures S4 and S5). By monitoring the decrease in the π–π* transition, the kinetics of the click reaction were analyzed (Figure 3a,b). It should be noted that the spectrum collection is focused on the 400–465 nm range to rapidly acquire spectra, which is essential for estimating reaction kinetics. The reaction rate constant under VSC at 298 K was determined to be 11.1 ± 1.5 × 10^–2^ (M^–1^ s^–1^), whereas the reaction rate constant without VSC was calculated to be 2.66 ± 0.43 × 10^–2^ (M^–1^ s^–1^), as shown in Figures S6 and S7. Thus, the reaction rate increased 4 times under VSC. The cavity mode was scanned around the absorption range of the C–H stretching vibrations (2800–3200 cm^–1^), and the reaction kinetics were maximized at the peak of the C–H stretching absorption (Figure 3c). By varying the temperature for the click reaction in the range of 288 and 318 K under VSC and non-VSC, the activation enthalpy (Δ*H* ^⧧^) and activation entropy (Δ*S* ^⧧^) were also evaluated by Eyring plots (Figure 4). The resulting values of Δ*H* ^⧧^ and Δ*S* ^⧧^ were summarized in Table 1. Since Δ*H* ^⧧^ under VSC is lower than that under non-VSC, this suggests that the relatively low energy facilitates the progression of the reaction. Additionally, Δ*S* ^⧧^ under VSC is also lower than that under non-VSC. The reduced Δ*S* ^⧧^ indicates that the molecular degrees of freedom decrease as the reaction progresses. The changes in both Δ*S* ^⧧^ and Δ*H* ^⧧^ may be attributed to a slight alteration in the assembled structures.

**Figure 3:**
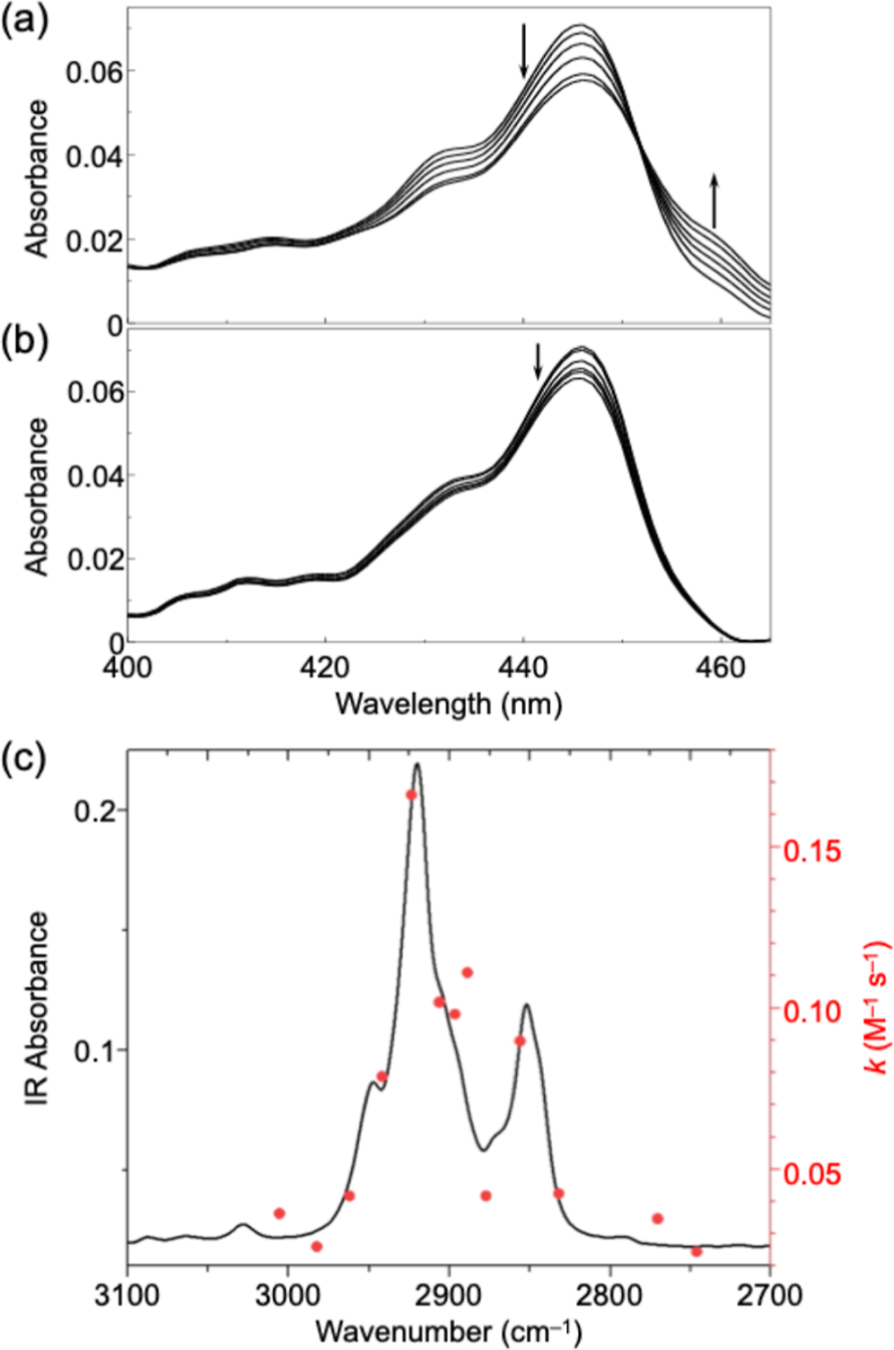
(a,b) Time-dependent UV–vis absorption spectra of **S­(NDI-1)** during the click reaction: (a) under VSC conditions of the C–H bond and (b) under non-VSC conditions, measured at time points of 2, 4, 6, 8, 11, and 13 min. (c) Click reaction rate constant *k* (M^–1^ s^–1^, red dots) plotted on the IR absorption spectrum of the solution (black line).

**Figure 4:**
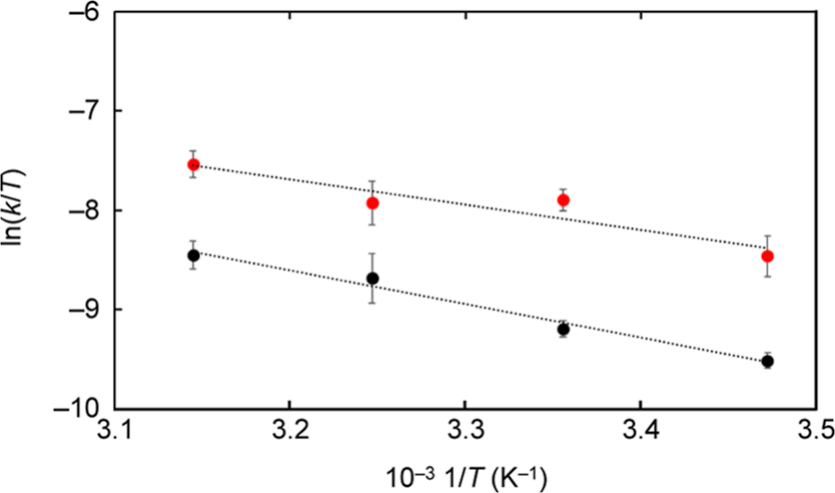
Eyring plots of click reactions under VSC (red dots) and non-VSC conditions (black dots).

**Table 1:** Thermodynamic Parameters: Activation Enthalpy (Δ*H* ^⧧^), Activation Entropy (Δ*S* ^⧧^)

state	Δ*H* ^⧧^ (kJ mol^–1^)	Δ*S* ^⧧^ (J mol^–1^)
non-VSC	28.1	–179
VSC	21.0	–194

To investigate the effect of VSC on reaction kinetics, we used a different molecule, **NDI-2**, composed of an ethynyl-attached NDI core and branched alkyl long chains at the imide positions (Scheme S1 and Figure S8). Unlike **NDI-1**, **NDI-2** does not form supramolecular assemblies and remains dispersed as monomers in solution, as confirmed by UV–vis absorption spectra and dynamic light scattering (DLS) measurements (Figure S9). Nonetheless, **NDI-2** can undergo an amino-yne click reaction between an ethynyl group and DEA to afford an amine monoadduct, **NDI-2–DEA**, as confirmed by ^1^H NMR spectra (Figures S1b and S10). The reaction rate constant under VSC was determined to be 20.2 ± 2.5 × 10^–2^ (M^–1^ s^–1^), whereas the reaction rate constant without VSC was calculated to be 15.2 ± 0.62 × 10^–2^ (M^–1^ s^–1^). The difference in rate constants between VSC and non-VSC conditions is only about 30%. Taking into account the standard errors in the measurements, VSC provides little to no significant improvement in the rate constants. Even though the cavity mode was scanned around the C–H stretching vibrations (2800–3200 cm^–1^), there was nearly no change in the reaction rate. These results suggest that VSC does not change the reactivity of the click reaction itself (Figure S11). Instead, the effect of VSC is likely linked to the change in the assembled structures of **S­(NDI-1)** because the reaction of monomers (**NDI-2**) is not sensitive to VSC. This result is reasonable, as the C–H stretch is not directly correlated with the click reaction.

### Emergence of Toroidal Assembly

Since the click reaction induces a transformation of assembled structures in **S­(NDI-1)**, atomic force microscopy (AFM) was performed after the click reaction to observe the resulting morphologies.^35^ The thick fibers appeared when the cavity mode was detuned from the C–H stretch, the same as the typical transformation of **S­(NDI-1)** fibers (Figures 5a,b and S12a–c).^31^ However, under the VSC of the C–H stretch, the transformation of **S­(NDI-1)** led to the formation of toroidal structures (Figures 5c,d). Although various NDI-based supramolecular structures have been created for decades, the formation of toroids had not been observed before.^31,36,37^ The toroidal structures were obtained irrespective of the substrates or spin-coating conditions (Figure S12). Scanning transmission electron microscopy of drop-cast samples further revealed toroidal morphologies (Figure S13). From these results, it can be deduced that the toroidal structures were formed in solution rather than during the spin-coating process on substrates. The AFM image analysis of 70 toroidal structures indicates that these toroids have a relatively uniform radius of 447 ± 76 nm (Figure S12c–g), with an average height of 16 nm (Figures 5d and S14a,b). Given that the height of the original fibers of **S­(NDI-1)** is approximately 2.0–2.5 nm (Figure S14c,d), the toroids are most likely formed by bundled fibers.

**Figure 5:**
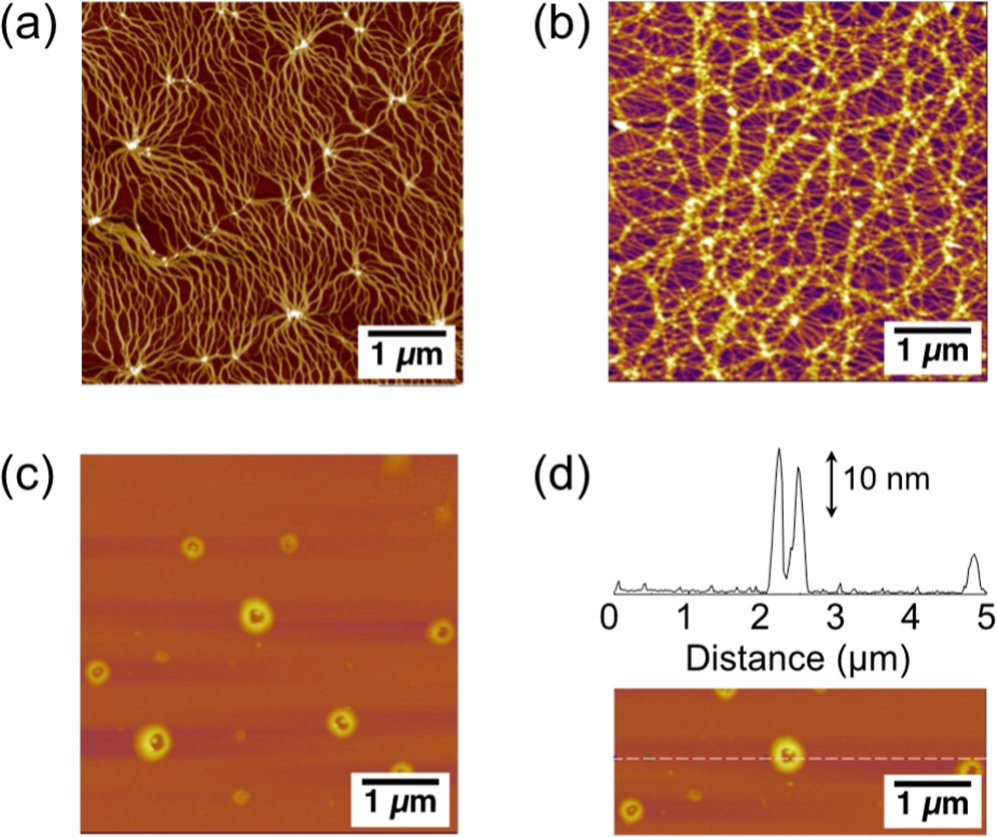
AFM height images of supramolecular polymers: (a) before the click reaction, (b) 60 min after the reaction initiation under non-VSC conditions, and (c) 60 min after the reaction initiation under VSC conditions. The reaction solution was diluted 10-fold, and it was spin-coated onto a silicon substrate. (d) Height profile of a typical toroidal structure along the white dotted line in the AFM image.

The toroids and fibers were analyzed by AFM–IR measurements, a technique that combines AFM with IR spectroscopy to achieve high spatial resolution for material characterization, particularly in the IR range. This technique enables the detection of molecular vibrations, making it useful for studying assembled structures on the nanoscale. By comparing the IR absorbance spectra of the toroids and fibers (Figure 6), we observed three distinct features in the toroidal structures: a shoulder around 1740 cm^–1^ (C=O asymmetric stretch), a peak at 1650 cm^–1^ (C=O symmetric stretch), and broadening around 1300–1400 cm^–1^ (C–H bending). These characteristic features suggest that the stacking of NDI cores and CH–CH alkyl interactions differs between toroids and fibers.

**Figure 6:**
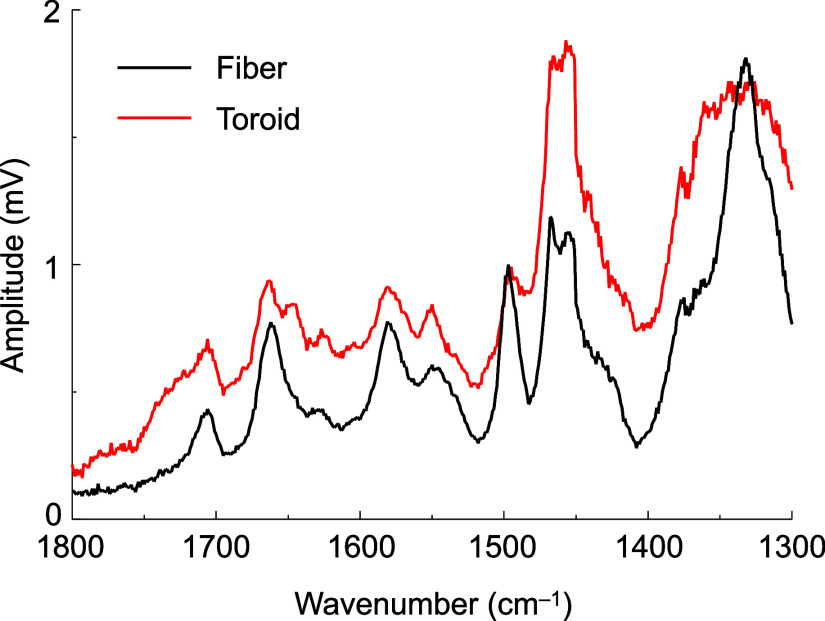
AFM–IR spectra of fibers (black) and toroids (red). The spectra represent averages of four measurement points.

To further investigate the influence of VSC on the assembled structures, we conducted a click reaction for **S­(NDI-1)** in a deuterated solvent mixture of MCH-*d*_14_/toluene-*d*_8_ (4:1 by volume). In an MCH/toluene mixture (4:1 by volume), the C–H stretching vibrations of **S­(NDI-1)**, MCH, and toluene are cooperatively coupled to a cavity mode. As a result, the effect of the VSC on **NDI-1** and the solvent molecules cannot be discussed separately. However, the C–H stretch of **S­(NDI-1)** around 2920 cm^–1^ does not overlap with the vibrational bands of MCH-*d*_14_ and toluene-*d*_8_ (Figure S15); thus, the effect of VSC only on solvent molecules can be observable in deuterated solvents. In deuterated solvents, fibrous structures were observed after the click reaction between **S­(NDI-1)** and DEA, even under the VSC of the C–D stretch (Figure S16). This result suggests that the structural transformation into toroidal assemblies is primarily attributed to the effect of the VSC on **NDI-1** rather than only on the solvent molecules. In other words, VSC likely modifies the interactions among **NDI-1** molecules, leading to a distinct transformation pathway that favors the formation of toroidal structures.

To elucidate the mechanism behind the formation of toroidal structures, theoretical simulations were performed to predict the stable packing states of the reaction product (denoted as **NDI-1–DEA**) in supramolecular structures. The transformation of these supramolecular structures occurred under the VSC conditions. After the reaction-induced transformation, the solution was extracted and spin-coated onto a silicon substrate to obtain AFM images. The presence of toroidal structures on silicon substrate means that the resulting toroidal structures are relatively stable, even under ambient conditions. Indeed, the toroidal structures remained unchanged after being kept under ambient conditions for several months or heated up to 333 K (Figure S17). Thus, the stable packing structures of **NDI-1–DEA** dimers were predicted using DFT with B3LYP-D3 ^38^/6–311++G­(d,p) in Gaussian 16 ^39^ and PM6-D3H4 ^40,41^ in MOPAC2016 ^42^ (see Supporting Information for details). The interaction between **NDI-1–DEA** is primarily driven by hydrogen bonding between amide groups, π–π interactions involving NDI units, and packing of alkyl chains. Simulations have proposed three distinct stable packing states for the **NDI-1–DEA** dimers (**entries-2**, **-3**, and **-4**; Figures S18 and S19 and Table S1). In **entry-2**, six alkyl chains extend directly above the NDI cores, obstructing further stacking of **NDI-1–DEA** and thereby hindering the formation of assembled structures. In contrast, **entries-3** and **-4** represent plausible stable configurations that are suitable for discussing the assembly of **NDI-1–DEA** (Figure 7).

**Figure 7:**
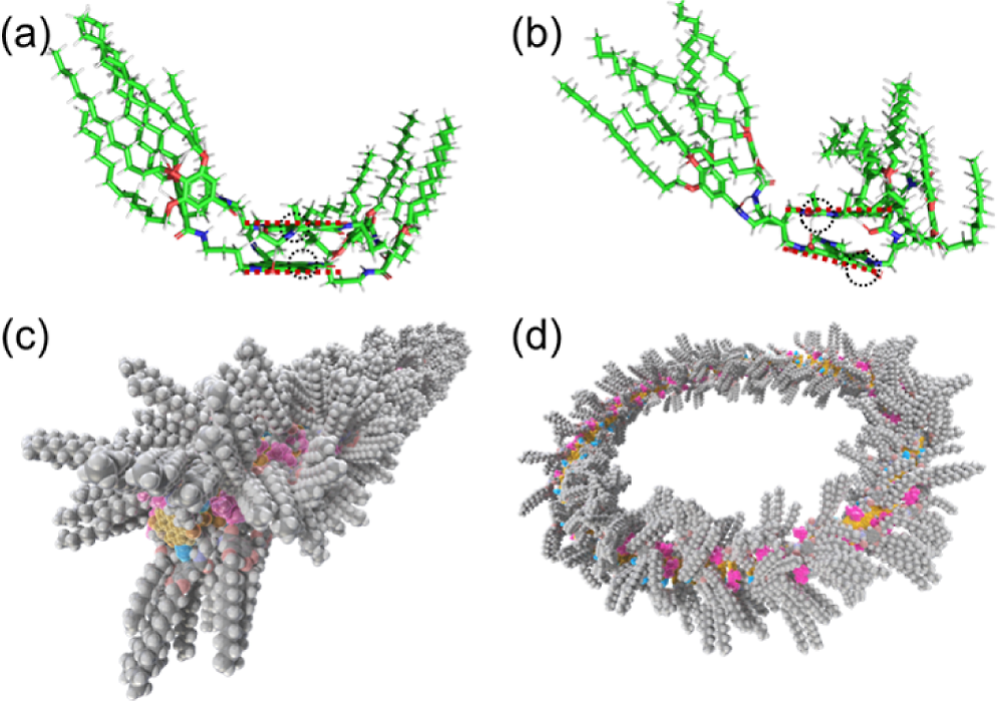
Optimized structures of **NDI-1** dimers: (a) **entry-3** and (b) **entry-4**. Carbon, hydrogen, oxygen, and nitrogen atoms are represented in green, white, red, and blue, respectively. Brown dotted lines denote the planes of the NDI cores. The dihedral angles of **entry-3** and **entry-4** are calculated to be 1.45° and 14.24°, respectively. Due to the tilted angles of adjacent NDI cores in **entry-4**, there is increased spacing between adjacent ethynyl groups (black dotted circles), facilitating click reactions. Using atomic coordinates from DFT calculations, assembled structures were modeled by repeating the dimer unit. Illustrative diagrams of the assembled structures corresponding to **entry-3** and **entry-4** are shown in (c) and (d), respectively. The near-planar NDI cores in **entry-3** lead to fiber-like assemblies, while the larger dihedral angle in **entry-4** promotes the formation of toroidal structures.

In **entry-3**, the NDI cores stack face-to-face, but in **entry-4**, the NDIs adopt a slightly slipped stacking configuration. Notably, the slipped stacking configuration in **entry-4** forms a dihedral angle of 14.24° (γ in Table S1), which contributes to the curvature required for the formation of toroidal assemblies. The structural extension derived from the simulated **NDI-1** leads to a one-dimensional assembly in **entry-3** and a curved assembly in **entry-4** (Figures 7c,d). These structural configurations suggest that **entry-3** and **entry-4** serve as the core assembled frameworks for fibers and toroids, respectively. While nothing definitive can be concluded at this stage, we are currently considering the following mechanisms based on our observations. The amine addition to **S­(NDI-1)** fibers initiates a transformation process in which curved NDI stacking assemblies give rise to shorter bundled fibers, ultimately leading to the formation of toroids under VSC. A secondary nucleation process may also play a significant role in the formation of these toroids, which are several hundred nanometers in diameter.^43^

Another unique feature of **entry-4** is the dihedral angle of NDI, which makes the space around the ethynyl groups more accessible to DEA. Comparing the reaction kinetics between monomers of **NDI-2** and supramolecular polymers of **S­(NDI-1)**, the monomers of **NDI-2** exhibit faster reaction kinetics than the supramolecular polymers of **S­(NDI-1)**. This difference arises from the increased steric hindrance at the ethynyl reactive sites in the stacked structures in the supramolecular polymers of **S­(NDI-1)**. The steric hindrance restricts the accessibility of amines to the ethynyl groups of **S­(NDI-1)**, slowing the reaction. In this work, under VSC, the click reaction within supramolecular polymers of **S­(NDI-1)** was accelerated, whereas no acceleration was observed in monomers of **NDI-2**. This result can be explained by the space around the ethynyl groups in **entry-4**, as the reduced steric hindrance in the supramolecular polymers under VSC promotes a faster click reaction. This acceleration was observed only in the supramolecular structures, explaining why the click reaction of **NDI-2** monomers was unaffected by VSC. These theoretically predicted stable configurations also provide hypothesized reasoning for the accelerated reactions within supramolecular polymers and the formation of toroidal structures.

The predicted structures of **entry-3** and **entry-4** align well with the AFM–IR spectra. In the toroidal assembly, the dihedral angle between the two NDI cores causes the C=O bond, farther from the hydrogen atom, to be positioned on one side. The presence of weak hydrogen bonds in some diimides results in a shoulder at 1740 cm^–1^ on the high-frequency side. Similarly, due to the dihedral angle, the C=O bond closer to the hydrogen atom is positioned on the opposite side. Strong hydrogen bonds in some diimides produce a peak at 1650 cm^–1^ on the low-frequency side. Additionally, the tilted overlap of the NDI molecules, induced by the dihedral angle, leads to various C–H states both inside and outside the ring, causing the observed broadening around 1300–1400 cm^–1^.

Additionally, the experimentally observed changes in Δ*H* ^⧧^ and Δ*S* ^⧧^ under VSC can be rationalized based on the predicted packing structures. In **entry-4**, the slipped configuration allows facile access of DEA to the ethynyl group, facilitating easier access of DEA to the ethynyl group, leading to a lower Δ*H* ^⧧^ under VSC. Furthermore, the enthalpy of the slipped stacking configuration in **entry-4** is likely higher than that of the face-to-face configuration in **entry-3**. Since the initial entropy under VSC is higher, the entropy changes during the click reaction can be more pronounced under VSC compared to those under non-VSC conditions.

The proposed structures can align with the effects of the VSC reported in previous studies. Under the VSC of the C–H stretching mode, the London dispersion forces between alkyl groups weaken, as observed by changes in the ^1^H NMR spectra.^24^ This reduced interaction between alkyl groups has also been identified in porphyrin supramolecular polymers, manifested as lower thermal stability at elevated temperatures. The simulation of packing structures in this work also suggests that the alkyl interactions in **entry-4** are weaker due to the slipped stacking configurations of **NDI-1–DEA**. This reduced interaction correlates with the calculated lower stability of **entry-4**. Considering previous studies, it is plausible that the VSC of the C–H stretch in solvent molecules and the alkyl groups of **NDI-1** induce weaker CH–CH interactions. This, in turn, facilitates the formation of **entry-4**, disclosing the unique toroidal assemblies herein.

## Conclusions

This work demonstrates the potential of VSC to manipulate molecules into unique assembly structures. The self-assembled fibers, stabilized by hydrogen bonding, π–π interactions, and alkyl chain interactions, underwent transformations of assembled structures upon the click reaction, which introduced tertiary amino groups into the assembly. Remarkably, the VSC of the C–H stretch significantly altered these transformations, leading to unique toroidal structures. Under VSC, reaction kinetics were enhanced compared to non-VSC conditions, as evidenced by a decrease in the π–π* transition band and the emergence of a charge transfer band in the UV–vis absorption spectra. The observed acceleration of reaction kinetics under VSC was specific to the supramolecular polymers, with nearly no enhancement detected for monomeric **NDI-2**, highlighting the dependence of VSC effects on assembled states. This suggests that the VSC influences the interplay of molecular interactions within the assembly.

AFM revealed a striking difference in the final structures formed under VSC. Unlike the thick fibers typically observed after the click reaction, VSC uniquely facilitated the formation of toroidal structures. Simulations provided insights into the plausible stacking configurations, showing that the weakened alkyl interactions and slipped packing create a curvature, enabling toroidal assembly. This configuration, with reduced steric hindrance around reactive sites, likely explains the enhanced reaction kinetics under the VSC.

The findings establish a link between the VSC-induced modulation of intermolecular interactions and the structural outcomes in supramolecular systems. By modulating intermolecular interactions and promoting alternative packing configurations, the VSC emerges as a unique tool to manipulate molecular assembly. These insights not only expand the understanding of VSC in complex molecular systems but also open avenues for creating assembled structures that are inaccessible through conventional methods.

## Supplementary Material



## Notes

Materials and methods, additional control experiments, and details of theoretical simulations (PDF)
