# Supplementary material for: Accessing a Hidden Pathway to Supramolecular Toroid through Vibrational Strong Coupling
Source: J Am Chem Soc. 2025 May 29;147(27):23528–35. doi: 10.1021/jacs.5c02960 (PMC12257502; doi:10.1021/jacs.5c02960)
Supplement: Supplementary file 1 [file ja5c02960_si_001.pdf]

Supporting Information

Accessing a Hidden Pathway to Supramolecular Toroid through Vibrational Strong Coupling

Shunsuke Imai,[‡1,2] Takumi Hamada,[‡3,4] Misa Nozaki,[5] Takatoshi Fujita,[5] Mariko Takahashi,[6] Yasuhiko Fujita,[6] Koji Harano,[7,8] Hiroshi Uji-i,[1,2,9,10] Atsuro Takai,*[3] and Kenji Hirai*[1,2]

‡ These authors contributed equally to this work.

1 Research Institute for Electronic Science (RIES), Hokkaido University, North 20 West 10, Kita ward, Sapporo, Hokkaido 001-0020, Japan.

2 Division of Information Science and Technology, Graduate School of Information Science and Technology, Hokkaido University, North 14 West 9, Kita ward, Sapporo, Hokkaido 060-0814, Japan.

3 Molecular Design and Function Group, National Institute for Materials Science (NIMS), 1-2-1 Sengen, Tsukuba, Ibaraki 305-0047, Japan.

4 Department of Materials Science and Engineering, Faculty of Pure and Applied Sciences, University of Tsukuba, 1-1-1 Tennodai, Tsukuba, Ibaraki 305-8577, Japan

5 Institute for Quantum Life Science, National Institutes for Quantum Science and Technology, 4-9-1 Anagawa, Inage-ku, Chiba 263-8555, Japan.

6 Research Institute for Sustainable Chemistry, National Institute of Advanced Industrial Science and Technology (AIST), Kagamiyama 3-11-32, Higashihiroshima, Hiroshima 739-0049, Japan.

7 Center for Basic Research on Materials, National Institute for Materials Science (NIMS), 1-2-1 Sengen, Tsukuba, Ibaraki 305-0044, Japan.

8 Research Center for Autonomous Systems Materialogy (ASMat), Institute of Integrated Research, Institute of Science Tokyo, 4259 Nagatsuda-cho, Midori-ku, Yokohama, Kanagawa 226-8501, Japan.

9 Department of Chemistry, KU Leuven, Celestijnenlaan 200F, Heverlee, Leuven 3001, Belgium.

10 Institute for Integrated Cell-Material Science (WPI-iCeMS), Kyoto University, Yoshida, Sakyo-ku, Kyoto 606-8317, Japan.

Table of Contents

Experimental Methods	Page S3
Supporting Data	
1. Amino-yne Click Reaction of **NDI-1** and **NDI-2** (**Figure S1**)	Page S8
2. Optical Cavities (**Figure S2**)	Page S9
3. Standard Curve for Absorption Coefficient (**Figure S3**)	Page S10
4. Time-dependent Absorption Spectra of **S(NDI-1)** during Amino-yne Click Reaction (**Figure S4**)	Page S11
5. Time-dependent ^{1}H NMR Spectra of **S(NDI-1)** during Amino-yne Click Reaction in Bulk Solution (**Figure S5**)	Page S13
6. IR Spectrum of Non-VSC Conditions (**Figure S6**)	Page S14
7. Fitting Analysis for Reaction Kinetics (**Figure S7**)	Page S15
8. Synthesis and Characterization of **NDI-2** (**Scheme S1** and **Figure S8**)	Page S16
9. **NDI-2** Dispersed in Monomeric State (**Figure S9**)	Page S18
10. Time-dependent ^{1}H NMR Spectra of NDI-2 during Amino-yne Click Reaction in Bulk Solution (**Figure S10**)	Page S20
11. Determination of Reaction Rate Constant of Amino-yne Click Reaction of **NDI-2** (**Figure S11**)	Page S21
12. AFM Images (**Figure S12**)	Page S22
13. Scanning Transmission Electron Microscopy of Fibers and Toroids (**Figure S13**)	Page S23
14. Height Profiles of Fibers and Toroids (**Figure S14**)	Page S24
15. VSC of Deuterated Solvents (**Figure S15**)	Page S25
16. AFM Images of Supramolecules after Click Reactions in Deuterated Solvents (**Figure S16**)	Page S26
17. Stability of Toroidal Structures (**Figure S17**)	Page S27
18. Theoretical Simulation of **NDI-1–DEA** dimers (**Figures S17**, **S18**, and **Table S1**)	Page S28
Supporting References	Page S31

Experimental Methods

Abbreviations:

MCH	methylcyclohexane
MCH-d_{14}	deutrated methylcyclohexane
$CHCl_3$	chloroform
$CDCl_3$	deutrated chloroform
CH_2Cl_2	dichloromethane
MeOH	methanol
DEA	diethylamine
VSC	vibrational strong coupling

Materials: MCH and toluene were purchased from FUJIFILM Wako Pure Chemical Corporation. MCH-d_{14} and toluene-d_8 were purchased from Sigma-Aldrich. The other reagents and solvents were purchased from Tokyo Chemical Industry, Sigma-Aldrich, FUJIFILM Wako Pure Chemical Corporation, or Kanto Chemical and used without further purification. Key compounds were purified by a recycling preparative HPLC (LaboACE LC-5060, Japan Analytical Industry) equipped with a gel permeation chromatography column (JAIGEL-2HH).

Nuclear Magnetic Resonance (NMR) Spectroscopy: NMR spectra were recorded on a JEOL JNM-ECZL600R spectrometer and analyzed using Mnova NMR software. Chemical shifts are presented in parts per million (ppm) relative to solvent peaks or tetramethylsilane as an internal standard.[S1]

Mass Spectrometry: High-resolution mass spectrometry was performed with a Bruker micrOTOF II spectrometer, equipped with an atmospheric pressure chemical ionization source (APCI TOF–MS).

UV–vis Absorption Spectroscopy for Bulk Solution: UV–vis absorption spectra of bulk solution were recorded on a JASCO V-730 spectrophotometer using a quartz cuvette. The time course of reactions was recorded with time-interval measurement mode.

Atomic Force Microscopy (AFM): The solution was extracted using a syringe after 60 min after the start of the click reaction and spin-coated at 3000 rpm for 60 seconds onto either silicon or highly oriented pyrolytic graphite (HOPG) substrates. The silicon substrates were sonicated in acetone twice for cleaning. Afterward, the substrates were removed from the acetone and completely dried under vacuum. To improve the dispersity of fibers or toroids, the reaction solution was typically diluted 10-fold before being spin-coated onto a silicon substrate. The differences in solution concentration are indicated in the respective figure legends. The resulting structures were analyzed by AFM in tapping mode using an MFP-3D-BIO-J system (Asylum Research – Oxford Instruments) or Dimension Icon (Bruker). A ScanAsyst cantilever (Bruker) was employed for the measurements. Toroidal structures obtained under VSC conditions were analyzed to gather statistical information. The size distribution of a total of 70 toroids extracted from different AFM images was obtained using ImageJ software.

Preparation of S(NDI-1): Synthesis of **NDI-1** was reported elsewhere.[S2] **S(NDI-1)** was prepared by slow cooling of **NDI-1** in MCH/toluene (4:1 by volume, 2 mM) from 363 K to 298 K at the rate of 1 K/min. We confirmed the appearance of a new absorption band at 446 nm attributed to **S(NDI-1)**. This band reached saturation near room temperature at the concentration of **NDI-1** used (2 mM), indicating that **NDI-1** was mostly converted to **S(NDI-1)**. We also confirmed by AFM that spin-coated samples of the resultant solution exhibited fibrous supramolecular polymers, as was observed previously at lower concentrations.[S2]

Fabrication of Optical Cavities: The cavity cell was manufactured by TTC Inc., Japan. Kapton film was sourced from DuPont Toray Specialty Materials K.K., and BaF_2 windows were obtained from Specac Ltd, UK. The BaF_2 windows were sputter-coated with a 150 nm thick indium tin oxide (ITO) layer. Two ITO-coated BaF_2 mirrors were positioned facing each

other, with the Kapton film placed between them, as illustrated in **Figure S2**. This mirror assembly was then inserted into the cavity cell. The mirror separation between the mirrors was precisely adjusted using micrometer-pitched screws (Mitsutoyo Corporation, Japan).

Monitoring Amino-yne Click Reaction: Before preparing the reaction solution, the FP cavity was set up and the mirrors were adjusted to be parallel, allowing the VSC or non-VSC of the C–H stretch to occur immediately after introducing the solution into the cavity. A solution of **S(NDI-1)** (2 mM in monomer unit) was prepared by mixing 60 μL of an MCH/toluene mixture (4:1 by volume) with 6 μL of a DEA solution (20 mM) in the same solvent mixture. The solutions were combined and mixed thoroughly by pipetting three times, marking this moment as time zero for estimating reaction kinetics. The prepared solution was then introduced into the cavity cell. The temperature of the cavity cell was controlled by a jacket with water flow system. Fourier transform infrared (FT-IR) spectroscopy was performed using a JASCO FT-IR 6800 to analyze the state of VSC. Subsequently, the absorbance of the π–π* transition was monitored with a JASCO V-750 spectrometer. The initial spectrum, covering the range of 400–465 nm, was recorded from 120 to 180 seconds after solution preparation, meaning that all experimental procedures—including solution introduction into cavity cell, and spectral acquisition of FT-IR and UV–vis absorption—were completed within 3 minutes. The absorption spectra were collected using a photomultiplier tube (PMT) detector with a scan rate of 200 nm/min. To enable rapid data acquisition, the collection range was focused on 400–465 nm, allowing spectra to be obtained within just 20 seconds.

The non-VSC experiments were conducted using the same FP cavity cell as in the VSC experiments. The only difference between VSC and non-VSC conditions is the distance between the mirrors, which determines the energy level of the cavity mode. Under non-VSC conditions, the cavity mode is not coupled to the CH stretch vibrational mode (**Figure S6**).

The amino-yne click reaction of **NDI-2** was monitored following the same procedures. To prepare the solution, 60 μL of an MCH/toluene mixture (4:1 by volume) containing **NDI-2** (2 mM) was combined with 6 μL of a DEA solution (20 mM) in the same solvent mixture.

Kinetics of Amino-yne Click Reaction: Amino-yne click reaction of **S(NDI-1)** or **NDI-2** with DEA (1 equivalent) follows the second-order reaction:

$$-\frac{d[A]}{dt} = k[A]^2$$

$$\frac{1}{[A]} = kt + \frac{1}{[A]_0}$$

where $[A]$ is the concentration of **S(NDI-1)**, **NDI-2**, and an amine, $[A]_0$ is their initial concentration, k is a reaction rate constant, and t is a reaction time.

$[A]$ was determined from the absorbance of the π–π* transition after correcting for the absorption coefficient in the optical cavity. A standard curve was generated by measuring solutions with varying concentrations (**Figure S3**).

Atomic Force Microscopy-Infrared Spectroscopy (AFM–IR): AFM–IR measurements were performed in the tapping AFM–IR mode, or heterodyne AFM–IR mode, with a commercial system (Dimension IconIR, Bruker). The measurements were performed with a gold-coated cantilever at 298 K with humidity below RH1%. The output of a quantum cascade laser (QCL) (Daylight, MIRCat) was pulsed at the frequency fm, where fm = f1 - f0. Here, f0 and f1 are the frequencies of the first and second resonances of the cantilever. In the measurements, f1 is used to drive the cantilever and the difference frequency at f0, generated by frequency mixing, was measured as an IR signal. The laser polarization was set to p-polarization (= parallel to the long axis of the probe). The details of the point measurement conditions are as follows: Pixel resolution = 1 cm^{-1}, Accumulation time = 60 s, Average = 3-5 times, Wavenumber range = 1800–1210 cm^{-1}. While visualizing the AFM images, nanoscale IR measurements were carried out on the fibrous and toroidal structures on the silicon substrate. The IR spectra were averaged over four measurement points for each structure.

Dynamic Light Scattering (DLS) Measurements: DLS data were obtained by using a Zetasizer Pro (Malvern Panalytical) equipped with 633 nm laser at 298 K. The scattering angle was kept at 173°.

Scanning Transmission Electron Microscopy (STEM): High-angle annular dark-field STEM, and bright-field STEM imaging were carried out on a Thermo Fisher Scientific Talos F200X G2 at an acceleration voltage of 80 kV. STEM images were recorded at a camera length of 410 mm (HAADF collection semi-angle: 25–150 mrad) with a probe current of ca 300 pA. STEM specimens were prepared by drop-casting sample solution onto thin carbon-coated copper grids (SHR-C075, Okenshoji Co.,Ltd.) followed by drying in vacuum.

Theoretical Calculation: The structural optimization of the **NDI-1–DEA** dimer was performed in several steps. First, the initial configurations of the NDI dimers were optimized at the level of B3LYP-D3[S3] with the 6-311++G(d,p) basis set using Gaussian 16,[S4] resulting in six suggested stacking structures. Next, the NDI dimer structures were optimized alongside ethynyl groups and DEA, where the side chains at imide positions were initially omitted. The optimization of this and the remaining steps were performed with the semiempirical PM6-D3H4[S5,S6] method using MOPAC.[S7] The optimized NDI dimers were combined with side chain structures, where the six alkyl chains were temporarily replaced with methoxy groups to simplify the process; the merged structures underwent further optimization. Finally, the methoxy groups were substituted back with the original alkyl chains, and the fully substituted structures were re-optimized.

The vibrational frequencies of the **NDI-1** monomer before and after the click reaction were also calculated using the PM6-D3H4 method. However, we did not observe a notable difference between them (results not shown).

Supporting Data

1. Amino-yne Click Reaction of NDI-1 and NDI-2

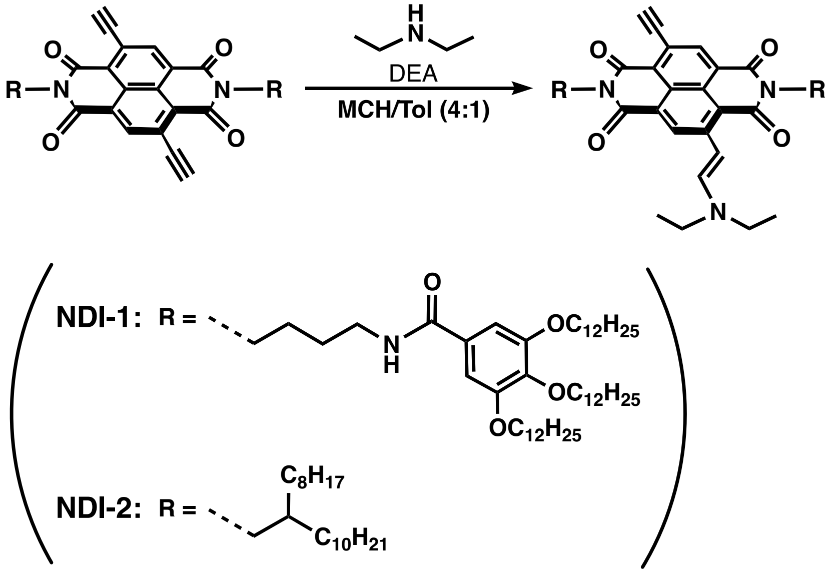

Figure S1. Chemical structures of (a) **NDI-1** and (b) **NDI-2** and their amino-yne click reactions with DEA to form amine monoadducts, **NDI-1–DEA** and **NDI-2–DEA**.

2. Optical Cavities

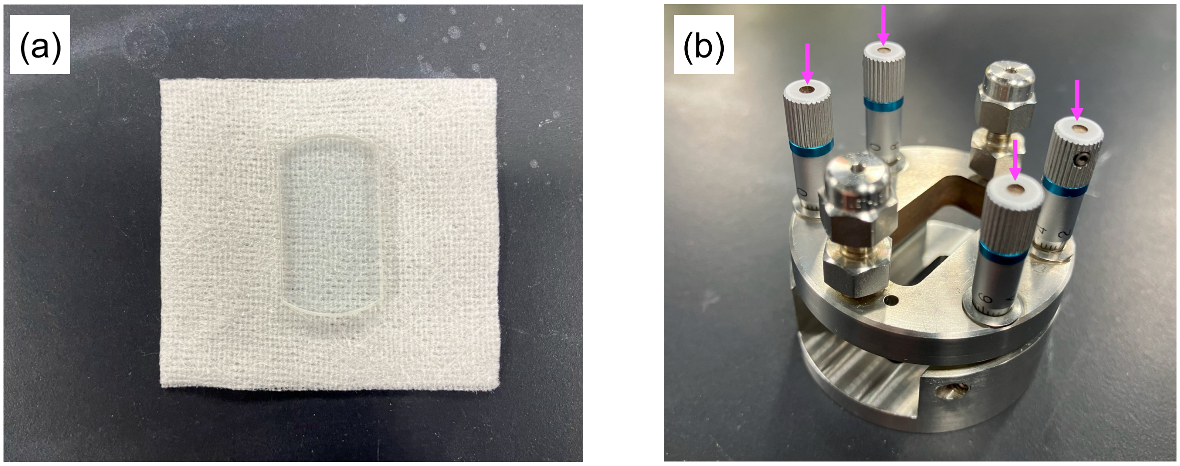

Figure S2. (a) Photograph of an ITO-coated BaF_2 mirror, which is transparent in the visible light region. (b) Photograph of the cavity cell, with arrows indicating the micrometer-pitched screws that enable rapid and precise tuning of the cavity modes.

3. Standard Curve for Absorption Coefficient

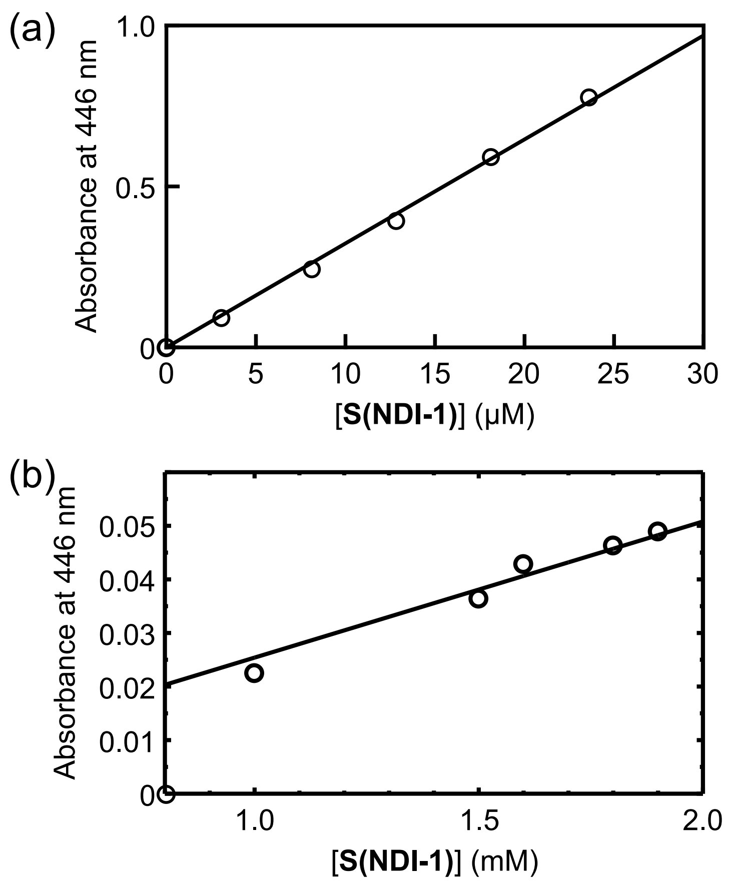

Figure S3. Standard curve of absorbance against solution concentration: (a) bulk solution in a 1 cm cuvette and (b) solution in an optical cavity with mirror separation of 10 μm. The gradients 3.23×10^4 M^{-1} cm^{-1} in (a) and 2.54×10^4 M^{-1} cm^{-1} in (b) correspond to the absorption coefficients of **S(NDI-1)**, indicating the absorption spectra in an optical cavity with ITO mirror can be measured reproducibly.

4. Time-dependent Absorption Spectra of S(NDI-1) during Amino-yne Click Reaction

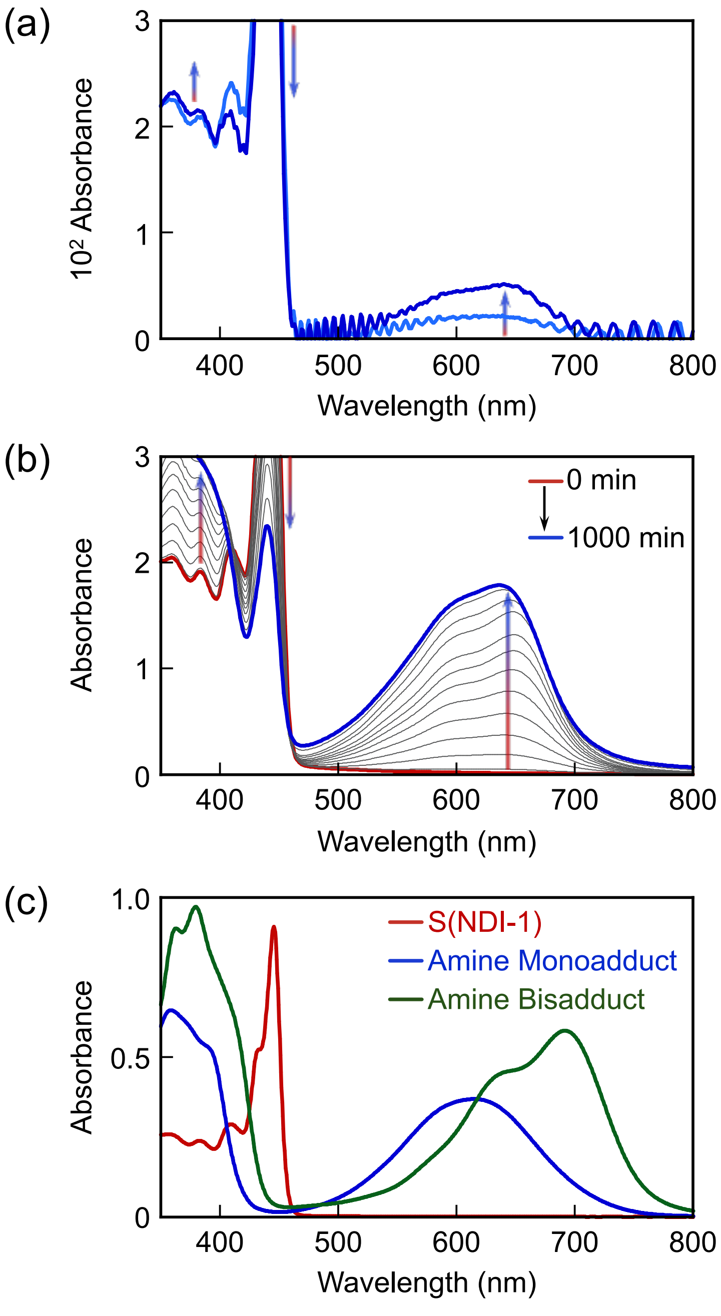

Figure S4. UV–vis absorption spectra during the click reaction between **S(NDI-1)** (2 mM in monomer unit) and DEA (2 mM) in MCH/toluene (4:1 by volume) at 298 K (a) in an FP cavity with the optical path length of 10 μm and (b) under bulk conditions with a 1 mm cuvette. In both cases, the absorbance of the π–π* transition at 446 nm decreased, while the

absorbance of charge-transfer increased over time. It should be noted that the kinetic analysis in an FP cavity focused solely on the 400–465 nm range to enable rapid acquisition and to avoid resonant peaks originating from cavity mode overtones, which is essential for accurately estimating the reaction kinetics. (c) UV–vis absorption spectra of **S(NDI-1)**, the corresponding amine monoadduct with DEA, and the bisadduct in bulk MCH/toluene (4:1 by volume, 25 μM in monomer unit) at 298 K, measured with a 1 cm cuvette. The absorption spectrum of the amine bisadduct is clearly different from that of the monoadduct, indicating that amine monoadduct is the main product under the conditions described in (a) and (b).

5. Time-dependent ^{1}H NMR Spectra of S(NDI-1) during Amino-yne Click Reaction in Bulk Solution

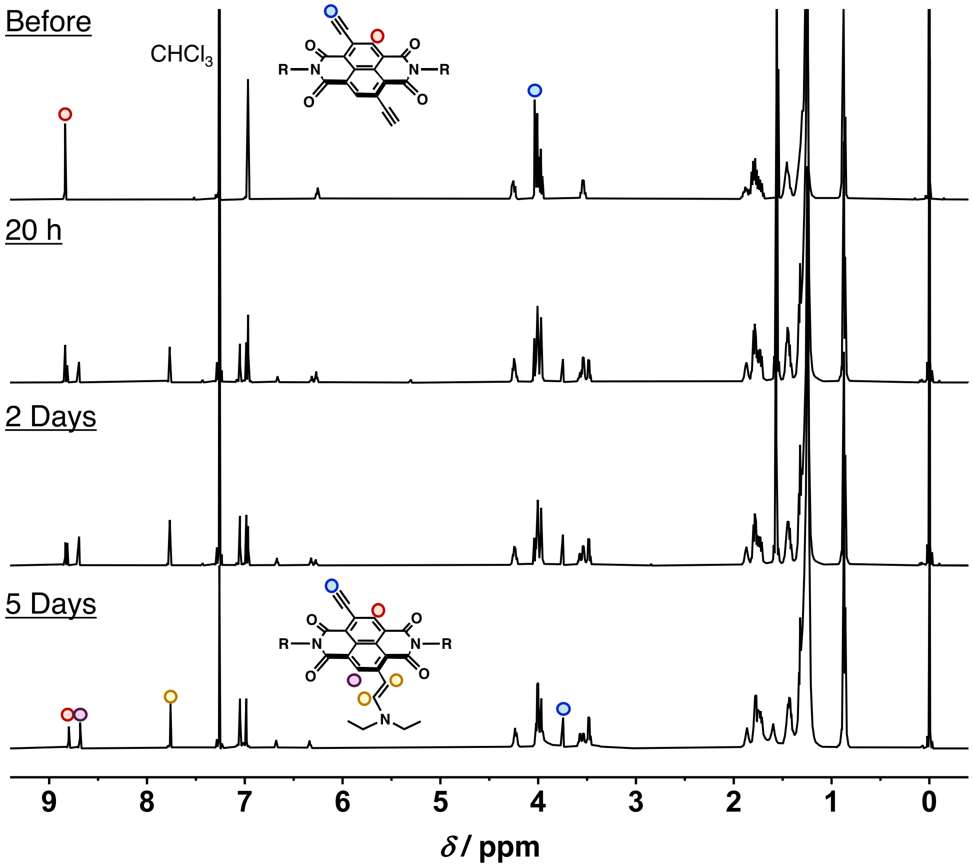

Figure S5. Time-dependent ^{1}H NMR spectra during the click reaction between **S(NDI-1)** (2 mM in monomer unit) and DEA (2 mM) in MCH/toluene (4:1 by volume) at 298 K. Because the ^{1}H NMR spectrum of the viscous, gelated solution of **S(NDI-1)** at 298 K in MCH-d_{14}/toluene-d_8 (4:1 by volume) showed no significant signals, the reaction solution was quenched with MeOH after a certain time to analyze the reaction product. The resulting precipitate was dried under vacuum, redissolved in $CDCl_3$ for ^{1}H NMR measurement at 298 K. Five days after the start of the click reaction, only an amine monoadduct was formed, with no corresponding bisadduct under the reaction conditions used, owing to the distinct deceleration of the second click reaction.[S10]

6. IR Spectrum of Non-VSC Conditions

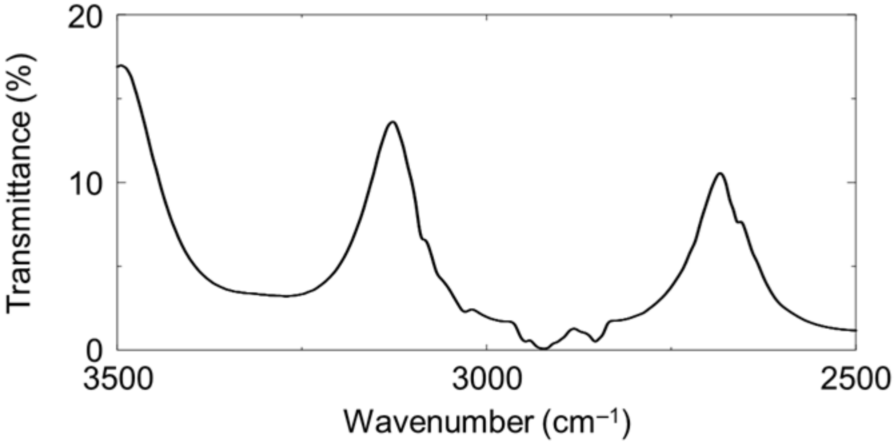

Figure S6. Fourier transform IR spectra of the solution introduced in an FP cavity. The cavity mode was detuned from 2908 cm^{-1} for non-VSC conditions.

7. Fitting Analysis for Reaction Kinetics

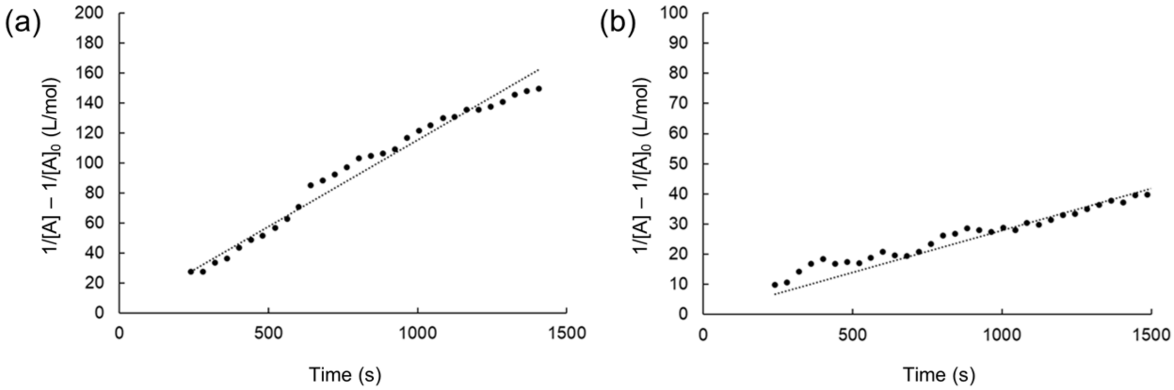

Figure S7. The fitting analysis to calculate the reaction rate constants for click reactions of **S(NDI-1)** under (a) VSC and (b) non-VSC conditions.

8. Synthesis and Characterization of NDI-2

Scheme S1. Synthetic scheme of **NDI-2**

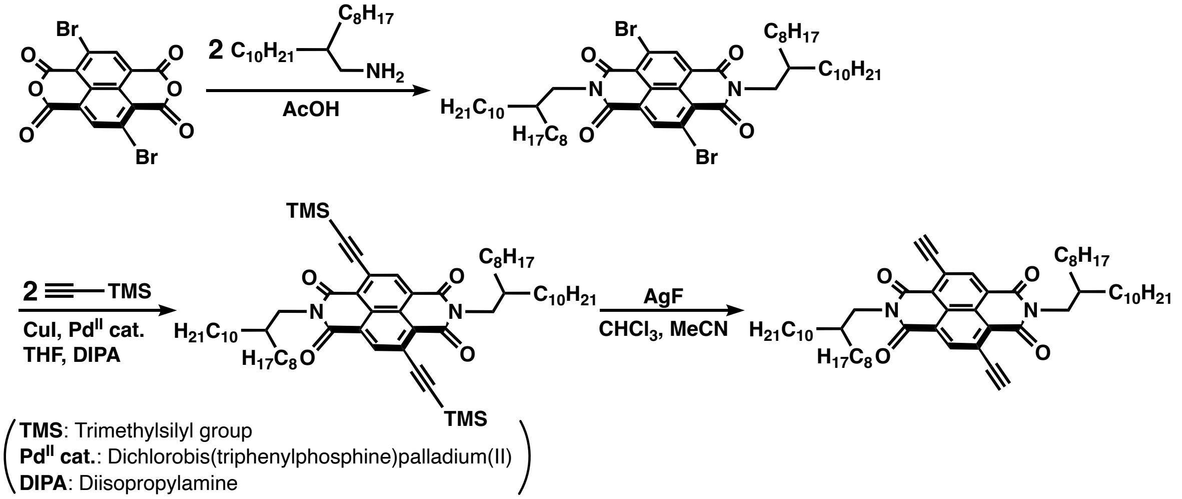

The syntheses of 2,6-dibromo-*N*,*N'*-bis(2-octyldodecyl)-1,8:4,5-naphthalenetetracarbox-diimide[S8, S9] and its conversion to TMS-protected ethynyl group of the bromo group by Sonogashira coupling[S10] were performed according to the literatures.

Subsequently, to a Schlenk flask containing TMS-protected ethynyl-attached NDI (317 mg, 0.31 mmol), silver(I) fluoride (AgF; 790 mg, 6.2 mmol) was added 40 mL of $CHCl_3$ and 10 mL of acetonitrile under Ar atmosphere. The brown mixture was stirred for 3 h at 298 K. After consumption of the starting material as indicated by thin-layer chromatography, 2 M HCl was added until the $CHCl_3$ phase became brown color. The crude was extracted with $CHCl_3$, and the organic phase was dried over Na_2SO_4, evaporated, and purified by silica gel column chromatography (CH_2Cl_2 and hexane = 1:1). The orange solid was further purified by reprecipitation from CH_2Cl_2 and MeOH to afford a yellow solid (134 mg, 49%). ^{1}H NMR (600 MHz, $CDCl_3$): δ (ppm) = 0.86 (m, 12H), 1.22–1.40 (m, 64H), 2.02 (m, 2H), 4.02 (s, 2H), 4.14 (d, J = 7.4 Hz, 4H), 8.85 (s, 2H). ^{13}C NMR (150 MHz, $CDCl_3$): δ (ppm) = 14.25, 22.80, 22.82, 26.48, 29.43, 29.48, 29.69, 29.73, 29.76, 29.78, 30.18, 31.65, 31.66, 32.02, 32.05, 36.54, 45.37, 82.23, 90.76, 125.54, 126.65, 126.68, 126.95, 138.10, 161.77, 162.06. APCI TOF–MS: calcd. for $C_{58}H_{86}N_2O_4$; $[M]^-$ 874.6588; found: 874.6564. See **Scheme S1** and **Figures S8**.

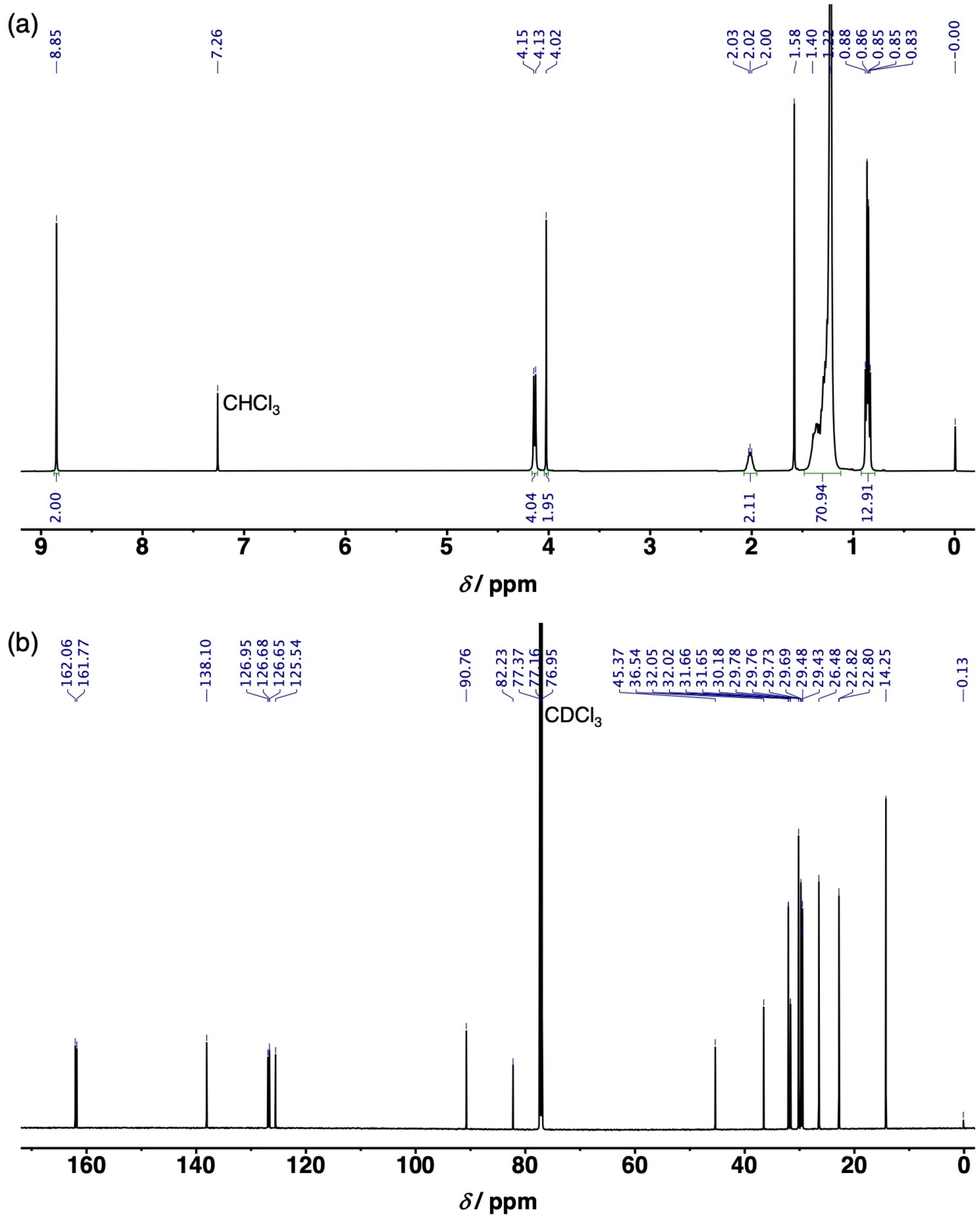

Figure S8. (a) ^{1}H and (b) ^{13}C NMR spectra of **NDI-2** in $CDCl_3$ at 298 K.

9. NDI-2 Dispersed in Monomeric State

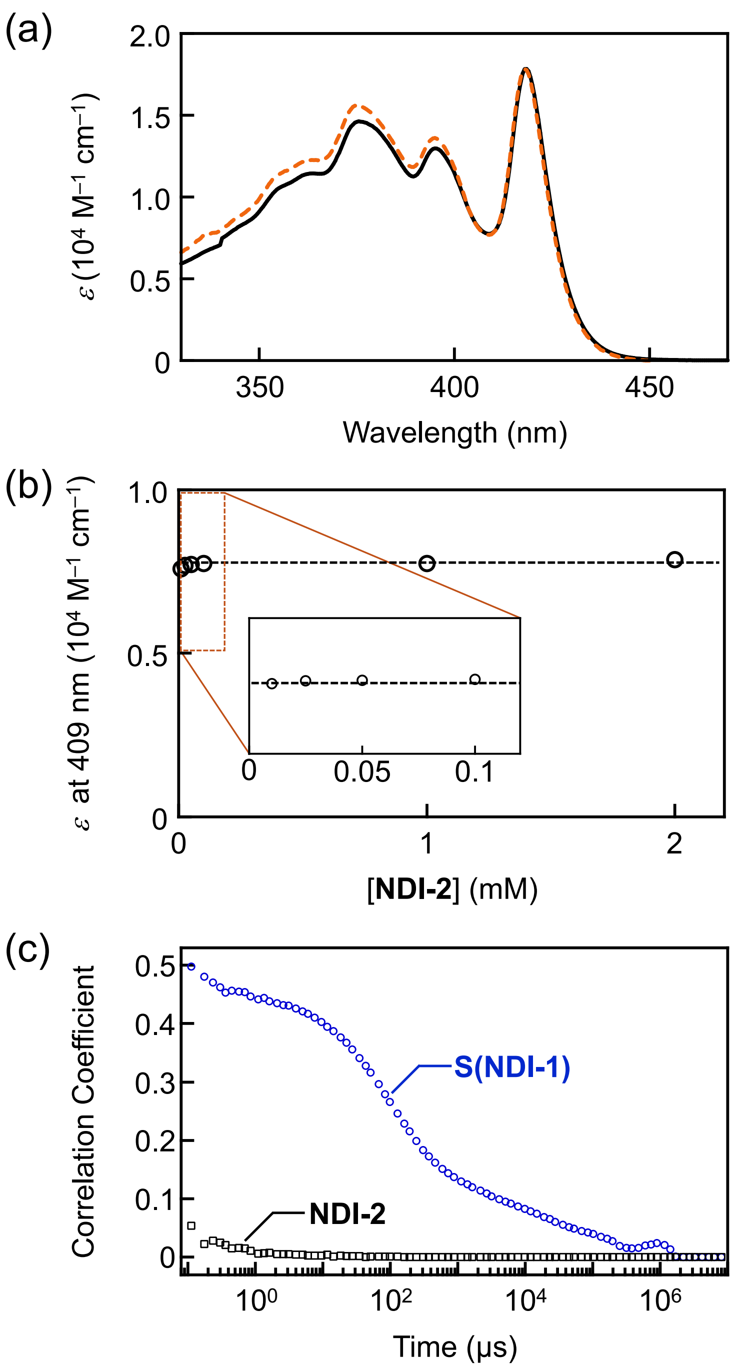

Figure S9. (a) UV–vis absorption spectrum of **NDI-2** at 1 mM (black line) and 25 μM (orange dashed line) in MCH/toluene (4:1 by volume) at 298 K. (b) The plot of absorption

coefficient (ε) at 409 nm against concentration of **NDI-2**. The ε values remained constant in the range of 10 μM to 2 mM, indicating no aggregation behavior of **NDI-2** under the experimental conditions. (c) The correlation functions of laser light scattering observed from **NDI-2** and **S(NDI-1)** in MCH/toluene (4:1 by volume, 2 mM) at 298 K. The distinct difference in relaxation times between **S(NDI-1)** and **NDI-2** indicates that **NDI-2** is dispersed in a monomeric state, while **S(NDI-1)** exists as a supramolecular polymer.

10. Time-dependent ^{1}H NMR Spectra of NDI-2 during Amino-yne Click Reaction in Bulk Solution

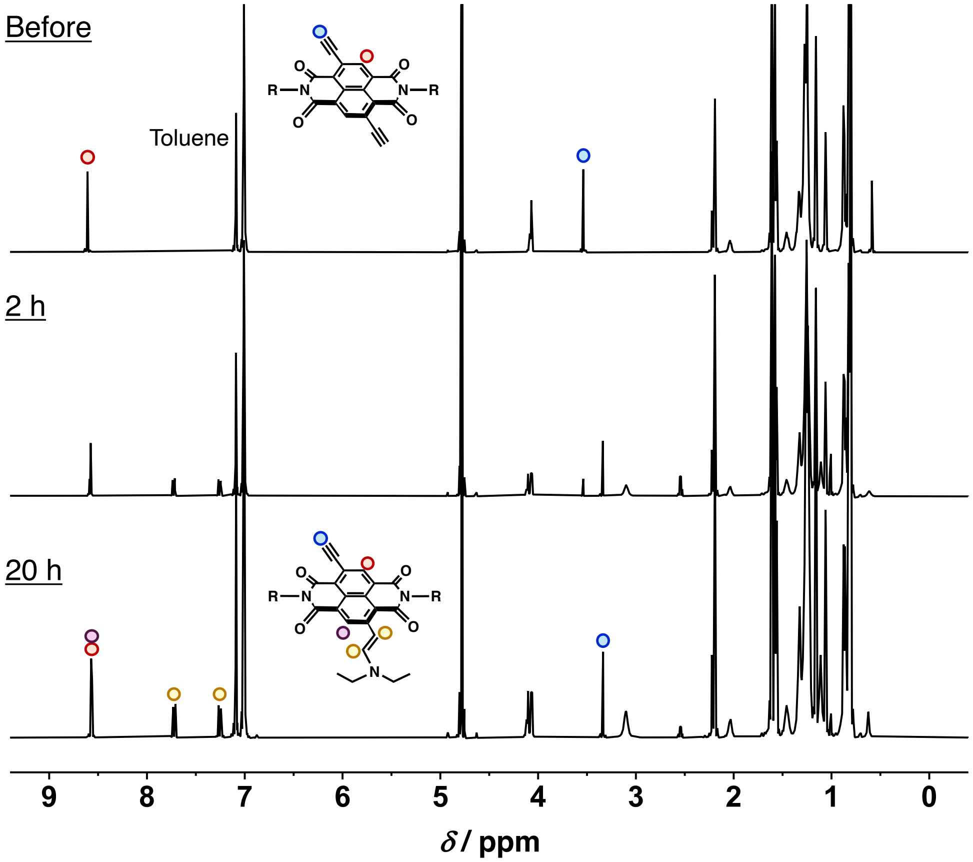

Figure S10. Time-dependent ^{1}H NMR spectra during the click reaction between **NDI-2** (2 mM) and DEA (2 mM) in MCH-d_{14}/toluene-d_8 (4:1 by volume) at 298 K. Only an amine monoadduct was formed, with no corresponding bisadduct under the reaction conditions used, owing to the distinct deceleration of the second click reaction.[S10] The sharp NMR signals of **NDI-2** in MCH-d_{14}/toluene-d_8 (4:1 by volume) at 298 K, in contrast to the barely visible signals of **S(NDI-1)**, also indicate that **NDI-2** is dispersed in a monomeric state.

11. Determination of Reaction Rate Constant of Amino-yne Click Reaction of NDI-2

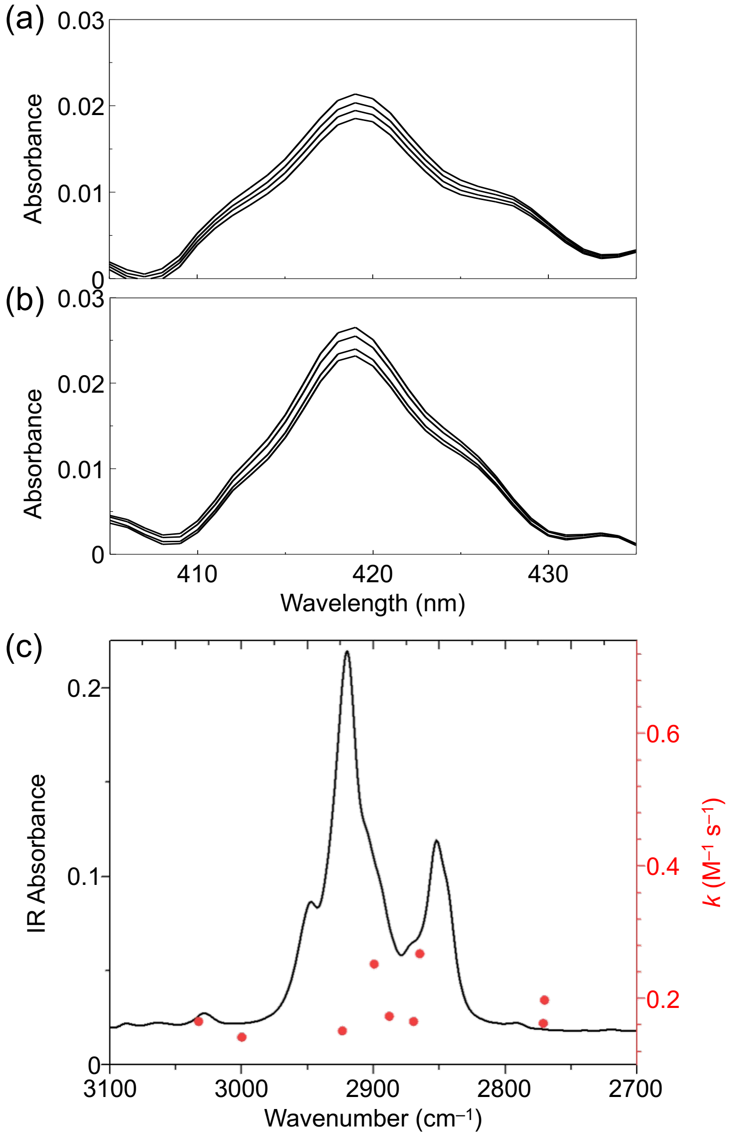

Figure S11. (a-b) Time-dependent UV–vis absorption spectra of **NDI-2** during amino-yne click reaction: (a) under VSC conditions of the C–H bond, and (b) under non-VSC conditions, measured at time points of 2, 4, 6, and 8 minutes. The absorbance of the π–π* transition of **NDI-2** is observed around 420 nm, which is lower than that of **S(NDI-1)**. This difference arises from slight variations in molecular structure and the fact that **NDI-2** remains dispersed in solution without forming assemblies. (c) Amino-yne click reaction rate constant k (M^{-1} s^{-1}, red dots) plotted on the IR absorption spectrum of the solution (black line).

12. AFM Images

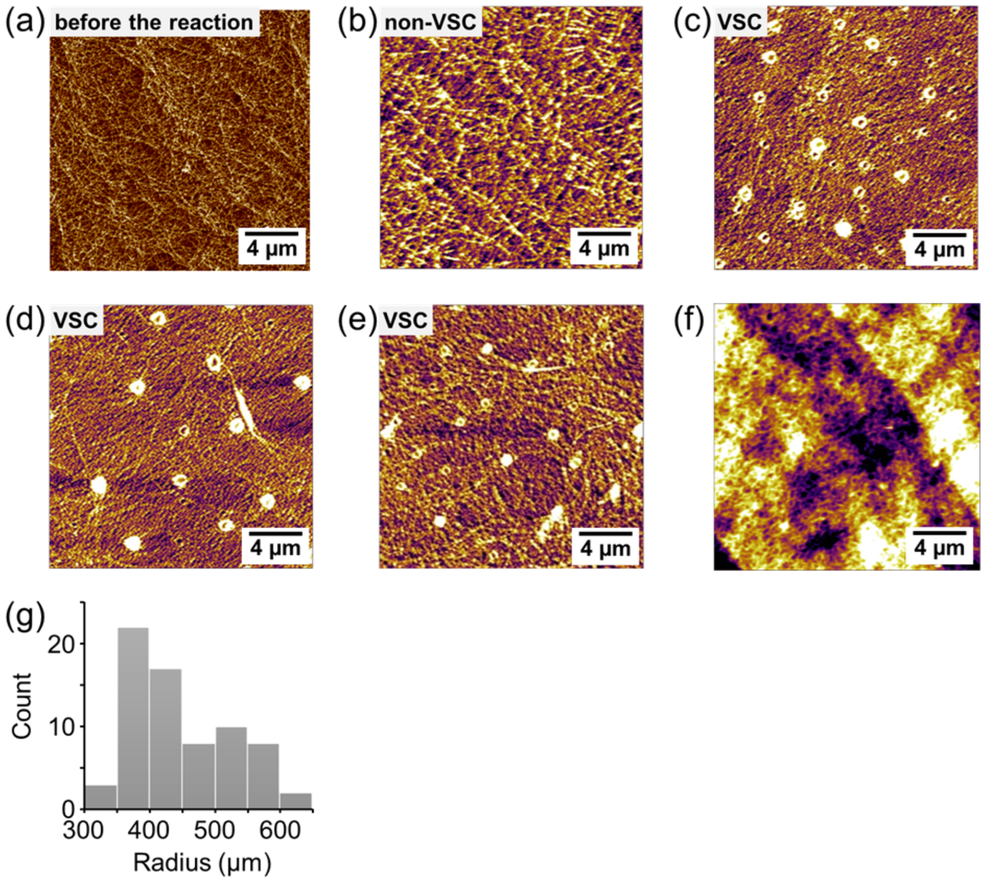

Figure S12. AFM height images of (a) supramolecular polymers before the click reaction; (b) after 60 min of the reaction initiation; and (c–e) after the reaction under VSC conditions. AFM images on (f) a silicon substrate and (g) highly ordered pyrolytic graphite (HOPG). (i) Statistical distribution of the radii of 70 observed toroidal structures.

13. Scanning Transmission Electron Microscopy of Fibers and Toroids

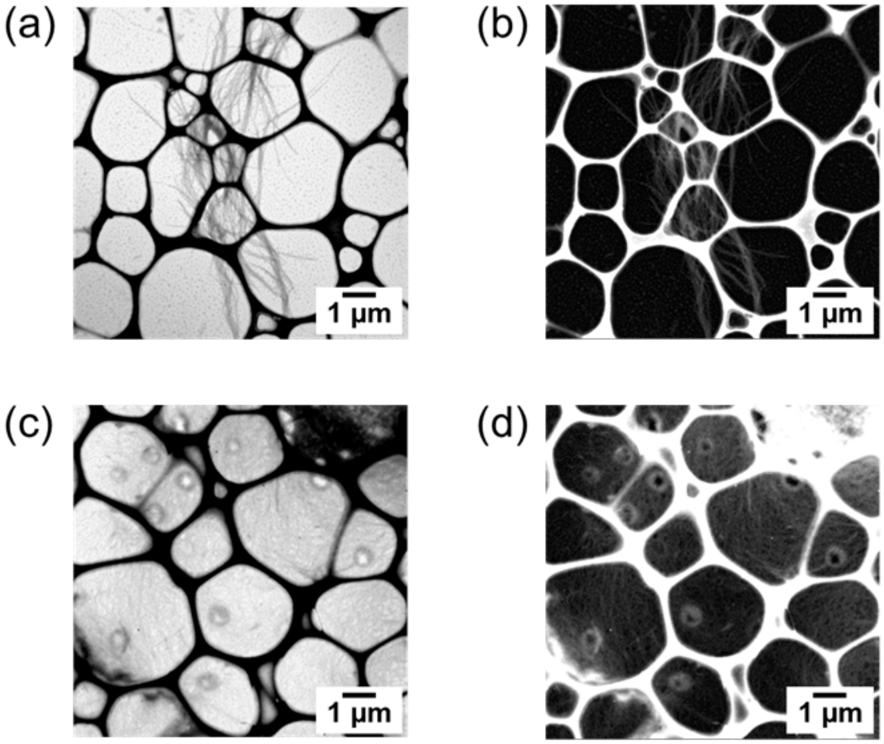

Figure S13. Bright-field STEM and high-angle annular dark-field STEM images of (a–b) fibers obtained under non-VSC conditions, and (c–d) toroids and fibers obtained under VSC conditions.

14. Height Profiles of Fibers and Toroids

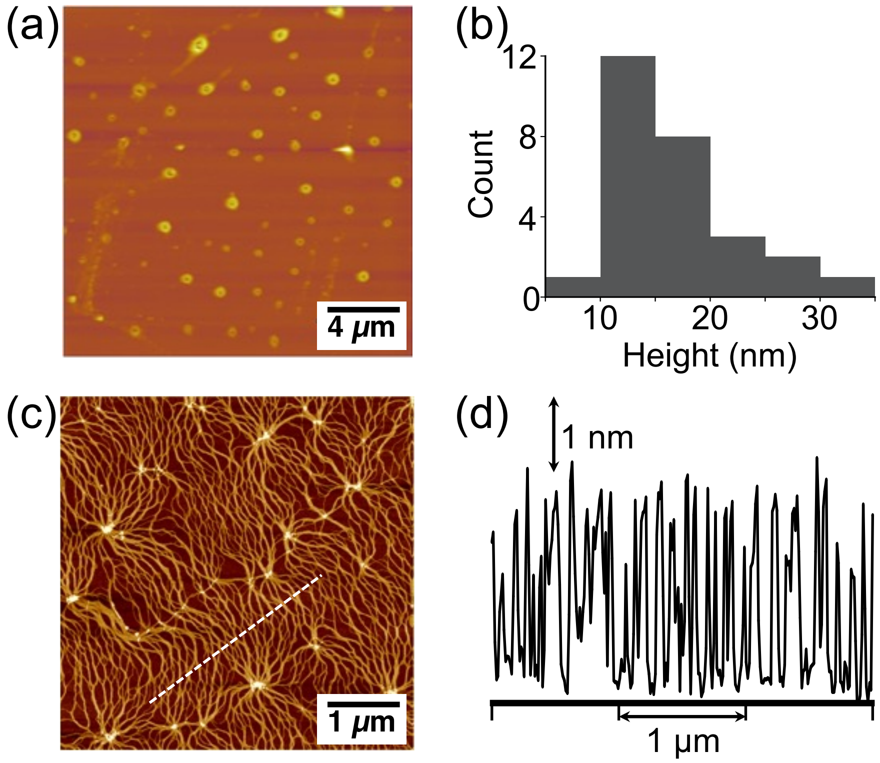

Figure S14. (a) AFM height image used for height profile analysis of toroidal structures. (b) Statistics of the height profile. (c–d) AFM height profile of the cross-section (white dashed line) of **S(NDI-1)**.

15. VSC of Deuterated Solvents

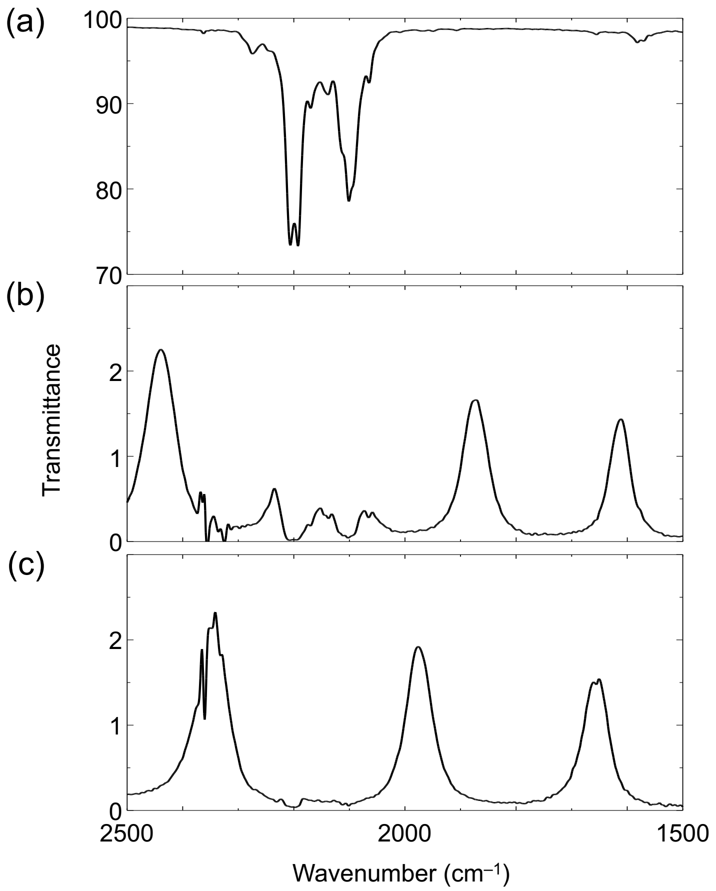

Figure S15. FT-IR spectra of (a) MCH-d_{14}/toluene-d_8 (4:1 by volume) solution containing **S(NDI-1)** and DEA, and (b–c) the same solution introduced into an FP cavity. (b) The cavity mode was tuned to 2132 cm^{-1} to achieve VSC, while (c) the cavity mode was detuned from the C–D stretch.

16. AFM Images of Supramolecules after Click Reactions in Deuterated Solvents

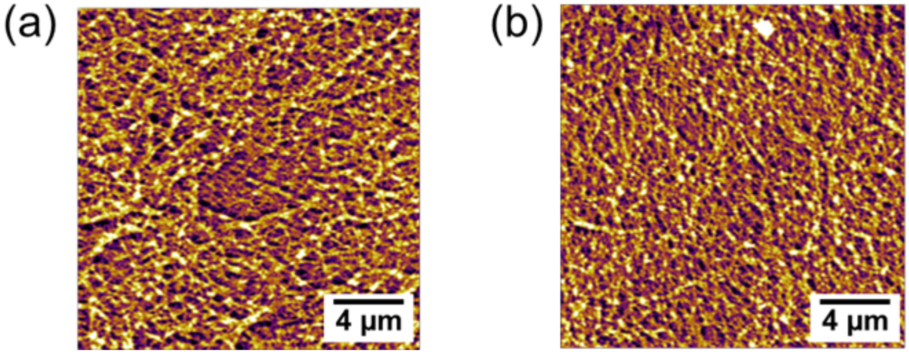

Figure S16. AFM height images of thick fibers after the reaction in MCH-d_{14} and toluene-d_8 (4:1 by volume) under (a) non-VSC and (b) VSC conditions.

17. Stability of Toroidal Structures

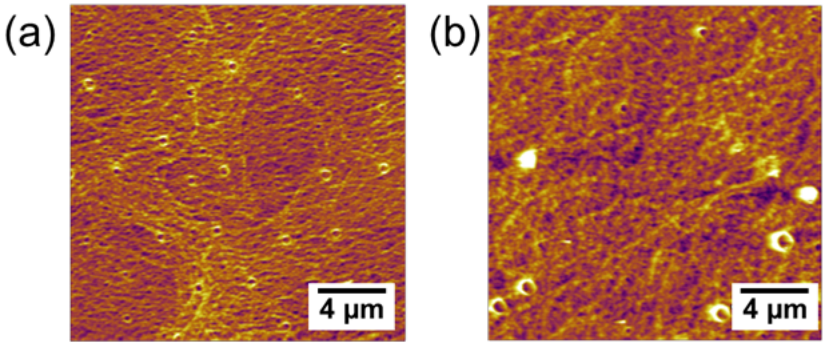

Figure S17. AFM images of toroidal structures after (a) being kept for several months under ambient conditions and (b) heating at 333 K for 30 minutes.

18. Theoretical Simulation of NDI-1–DEA dimers

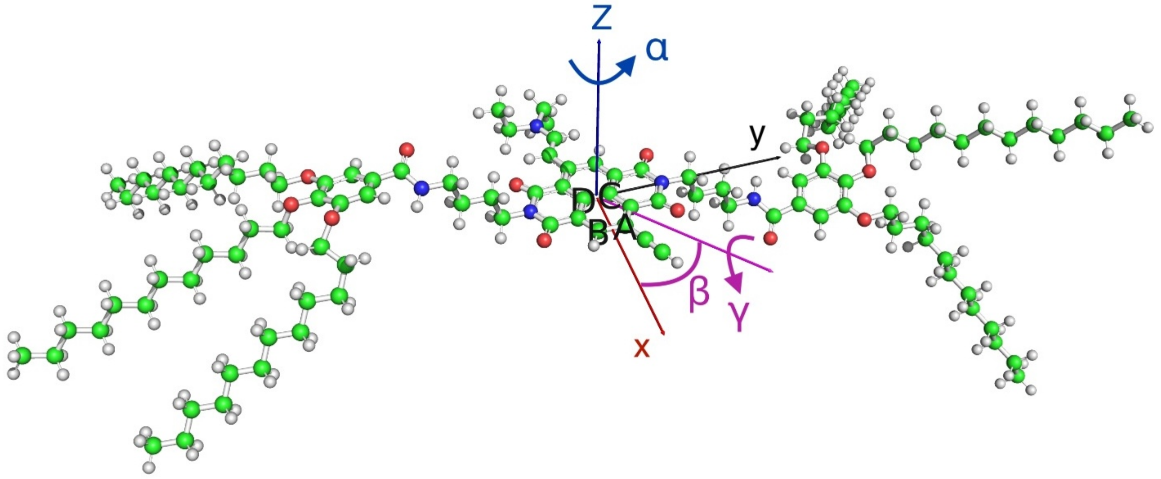

Figure S18. The definitions of structural parameters.

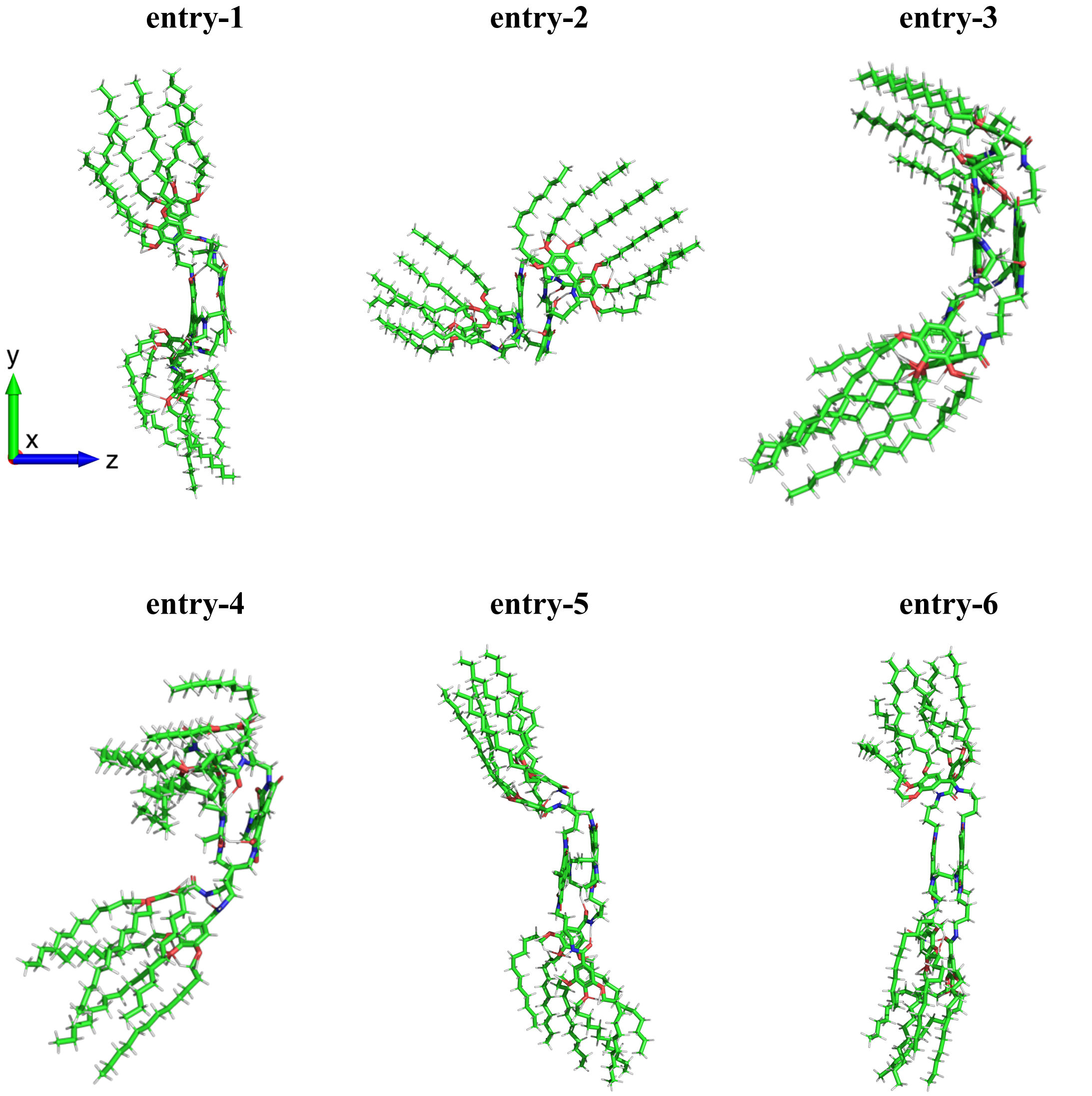

Figure S19. The optimized structures of **NDI-1–DEA** dimers.

Table S1. The structural parameters for optimized structures of **NDI-1–DEA** dimers shown in **Figure S18**

	X (Å)	Y (Å)	Z (Å)	β (°)	γ (°)	α (°)	heat of formation energy (kJ mol^{-1})
**entry-1**	–0.50	1.11	3.49	44.84	–5.87	–131.27	–5231.555
**entry-2**	–0.23	–3.44	3.21	–19.56	–165.92	81.58	–5304.456
**entry-3**	–0.28	–1.46	3.39	172.93	–1.45	139.97	–5295.868
**entry-4**	–2.63	–0.97	3.63	–177.50	–14.24	160.43	–5295.808
**entry-5**	–1.50	–2.13	–3.60	–36.79	–169.52	86.60	–5195.743
**entry-6**	–1.83	1.18	3.41	–63.60	–176.18	60.82	–5226.651

These parameters are defined by a series of operations that align the atoms of one **NDI-1–DEA** molecule (labeled as A, B, C, D in **Figure S11**) with the corresponding atoms of another **NDI-1–DEA** molecule. These operations consist of a rotation of α degrees around the z-axis, a rotation of γ degrees around an axis defined by (cos α, cos β, 0), and a translation by (X, Y, Z).

Entries-2–4 represent stable structures of **NDI-1–DEA** dimers. In **entry-2**, six alkyl chains extend directly above the NDI cores, which can hinder further stacking of **NDI-1–DEA** and prevent the formation of assembled structures. As a result, **entries-3** and **-4** are plausible structures for discussing the most stable and metastable configurations of **NDI-1–DEA**.

Supporting References

(S1) Fulmer, G. R.; Miller, A. J. M.; Sherden, N. H.; Gottlieb, H. E.; Nudelman, A.; Stoltz, B. M.; Bercaw, J. E.; Goldberg, K. I. NMR Chemical Shifts of Trace Impurities: Common Laboratory Solvents, Organics, and Gases in Deuterated Solvents Relevant to the Organometallic Chemist. *Organometallics* **2010**, *29*, 2176–2179.

(S2) Tan, M.; Takeuchi, M.; Takai, A. Spatiotemporal dynamics of supramolecular polymers by *in situ* quantitative catalyst-free hydroamination. *Chem. Sci.* **2022**, *13*, 4413–4423.

(S3) Grimme, S.; Ehrlich, S.; Goerigk, L. Effect of the damping function in dispersion corrected density functional theory. *J. Comput. Chem.* **2011**, *32*, 1456–1465.

(S4) Frisch, M. J.; Trucks, G. W.; Schlegel, H. B.; Scuseria, G. E.; Robb, M. A.; Cheeseman, J. R.; Scalmani, G.; Barone, V.; Petersson, G. A.; Nakatsuji, H.; Li, X.; Caricato, M.; Marenich, A. V.; Bloino, J.; Janesko, B. G.; Gomperts, R.; Mennucci, B.; Hratchian, H. P.; Ortiz, J. V.; Izmaylov, A. F.; Sonnenberg, J. L.; Williams-Young, D.; Ding, F.; Lipparini, F.; Egidi, F.; Goings, J.; Peng, B.; Petrone, A.; Henderson, T.; Ranasinghe, D.; Zakrzewski, V. G.; Gao, J.; Rega, N.; Zheng, G.; Liang, W.; Hada, M.; Ehara, M.; Toyota, K.; Fukuda, R.; Hasegawa, J.; Ishida, M.; Nakajima, T.; Honda, Y.; Kitao, O.; Nakai, H.; Vreven, T.; Throssell, K.; Montgomery, J. A., Jr.; Peralta, J. E.; Ogliaro, F.; Bearpark, M. J.; Heyd, J. J.; Brothers, E. N.; Kudin, K. N.; Staroverov, V. N.; Keith, T. A.; Kobayashi, R.; Normand, J.; Raghavachari, K.; Rendell, A. P.; Burant, J. C.; Iyengar, S. S.; Tomasi, J.; Cossi, M.; Millam, J. M.; Klene, M.; Adamo, C.; Cammi, R.; Ochterski, J. W.; Martin, R. L.; Morokuma, K.; Farkas, O.; Foresman, J. B.; Fox, D. J. *Gaussian 16 Revision C.01*, Gaussian, Inc., Wallingford CT, **2016**.

(S5) Stewart, J. J. P. Optimization of parameters for semiempirical methods V: Modification of NDDO approximations and application to 70 elements. *J. Mol. Model.* **2007**, *13*, 1173–1213.

(S6) Řezáč, J.; Hobza, P. Advanced Corrections of Hydrogen Bonding and Dispersion for Semiempirical Quantum Mechanical Methods. *J. Chem. Theory Comput.* **2012**, *8*, 141–151.

(S7) Stewart, J. J. P. *MOPAC2016*, Stewart Computational Chemistry, Colorado Springs, CO, USA http://OpenMOPAC.net (accessed May, 2025).

(S8) Kudla, C. J.; Dolfen, D.; Schottler, K. J.; Koenen, J.-M.; Breusov, D.; Allard, S.; Scherf, U. Cyclopentadithiazole-Based Monomers and Alternating Copolymers. *Macromolecules* **2010**, *43*, 7864–7867.

(S9) Royakkers, J.; Guo, K. P.; Toolan, D. T. W.; Feng, L.-W.; Minotto, A.; Congrave, D. G.; Danowska, M.; Zeng, W. X.; Bond, A. D.; Al-Hashimi, M.; Marks, T. J.; Facchetti, A.; Cacialli, F.; Bronstein, H. Molecular Encapsulation of Naphthalene Diimide (NDI) Based π-Conjugated Polymers: A Tool for Understanding Photoluminescence. *Angew. Chem. Int. Ed.* **2021**, *60*, 25005–25012.

(S10) Takai, A.; Takeuchi, M. Catalyst-Free Reaction of Ethynyl-π-Extended Electron Acceptors with Amines. *Bull. Chem. Soc. Jpn.* **2018**, *91*, 44–51.
